# Optimizing the Use of Zebrafish Feeding Trials for the Safety Evaluation of Genetically Modified Crops

**DOI:** 10.3390/ijms20061472

**Published:** 2019-03-23

**Authors:** Isabelle J. Gabriëls, Lucia Vergauwen, Marthe De Boevre, Stefan Van Dongen, Ronny Blust, Sarah De Saeger, Mia Eeckhout, Marc De Loose, Dries Knapen

**Affiliations:** 1 Zebrafishlab, Veterinary Physiology and Biochemistry, Department of Veterinary Sciences, University of Antwerp, Universiteitsplein 1, 2610 Wilrijk, Belgium; isabelle.gabriëls@uantwerpen.be (I.J.G.); lucia.vergauwen@uantwerpen.be (L.V.); 2 Systemic Physiological and Ecotoxicological Research (SPHERE), Department of Biology, University of Antwerp, Groenenborgerlaan 171, 2020 Antwerpen, Belgium; ronny.blust@uantwerpen.be; 3 Centre of Excellence in Mycotoxicology and Public Health, Department of Bioanalysis, Ghent University, Ottergemsesteenweg 460, 9000 Gent, Belgium; marthe.deboevre@ugent.be (M.D.B.); sarah.desaeger@ugent.be (S.D.S.); 4 Evolutionary Ecology Group, Department of Biology, University of Antwerp, Universiteitsplein 1, 2610 Wilrijk, Belgium; stefan.vandongen@uantwerpen.be; 5 Department of Food Technology, Food safety and Health, Faculty of Bioscience Engineering, Ghent University, Valentin Vaerwyckweg 1, 9000 Gent, Belgium; mia.eeckhout@ugent.be; 6 Technology and Food Sciences Unit, Institute for Agricultural and Fisheries Research (ILVO), Burg. Van Gansberghelaan 115, 9820 Merelbeke, Belgium; marc.deloose@ilvo.vlaanderen.be

**Keywords:** food safety, genetically modified crops, zebrafish, feeding trial

## Abstract

In Europe, the toxicological safety of genetically modified (GM) crops is routinely evaluated using rodent feeding trials, originally designed for testing oral toxicity of chemical compounds. We aimed to develop and optimize methods for advancing the use of zebrafish feeding trials for the safety evaluation of GM crops, using maize as a case study. In a first step, we evaluated the effect of different maize substitution levels. Our results demonstrate the need for preliminary testing to assess potential feed component-related effects on the overall nutritional balance. Next, since a potential effect of a GM crop should ideally be interpreted relative to the natural response variation (i.e., the range of biological values that is considered normal for a particular endpoint) in order to assess the toxicological relevance, we established natural response variation datasets for various zebrafish endpoints. We applied equivalence testing to calculate threshold equivalence limits (ELs) based on the natural response variation as a method for quantifying the range within which a GM crop and its control are considered equivalent. Finally, our results illustrate that the use of commercial control diets (CCDs) and null segregant (NS) controls (helpful for assessing potential effects of the transformation process) would be valuable additions to GM safety assessment strategies.

## 1. Introduction

The safety of genetically modified (GM) crops developed for food and/or feed use has been questioned by the general public ever since their introduction in the late 90s. The development of regulatory frameworks for the assessment and use of GM crops has evolved in different directions throughout the years in different parts of the world. In general, a number of countries (e.g., USA, Canada, Argentina, etc.) apply a product-based approach, in which regulatory requirements depend on the properties of the final GM crop irrespective of the methods by which it was developed [1]. Other legislations (e.g., in the European Union (EU), China, India, etc.) require a more rigorous process-oriented approach, where regulation is based on the technology used to create these crops [1,2,3]. In Europe, socio-economic concerns on food safety in general, and the use of GM crops as food/feed products in particular, has resulted in a significant politicization of the regulatory framework of GM crops [4] and ultimately a more stringent scientific risk assessment carried out by the European Food Safety Agency (EFSA, Regulation EC No 1829/2003). The EFSA GM crop assessment procedure consists of four main steps: (1) the molecular characterization of the GM crop, (2) the comparative analysis of compositional, phenotypic and agronomic properties, (3) the safety assessment for humans and animals concerning the nutritional value, potential allergenic and/or toxic effects and (4) the safety assessment for the environment. In step 3, the potential toxicity of the GM crop is evaluated using animal feeding trials. However, no standardized feeding trial protocols currently exist that are specifically dedicated to the testing of GM crops. The EFSA therefore published a guidance document for the toxicological evaluation of GM crops as whole food/feed, which is based on the use of an adapted version of a 90-day oral toxicity study in rodents (OECD, TG 408 [5]; EFSA [6]; Regulation EC No 1829/2003). Initially, the EFSA advised to consider carrying out a 90-day rodent feeding trial on a case-by-case approach—that is, only in cases where one of the preceding analysis steps (e.g., the compositional analysis) demonstrated substantial differences between the GM crop and its conventional counterpart. However, since the adoption of the EU Commission Implementation Regulation on applications for authorization of genetically modified food and feed in 2013 (European Commission, No. 503/2013), the 90-day rodent feeding trial using whole GM food/feed became a mandatory part of GM crop safety assessment. Ever since, the justification for routine GM crop testing using whole food feeding trials has been debated by several stakeholders [7,8,9,10], and efforts have been made by European authorities to evaluate the added value and limitations of these animal studies. Two key projects that received funding within the Seventh Framework Program of the European Commission are GMO Risk Assessment and Communication of Evidence (GRACE) in 2012 [11] and GM Plant Two Year Safety Testing (G-TwYST) in 2014 [12]. Based on the outcome of these projects it has been suggested that routine animal toxicity testing of GM whole food/feed does not provide relevant additional information on potentially unintended effects in the absence of concerns based on any of the earlier safety assessment steps. However, in 2017, the European Commission concluded: “Difficulties remain to define, with the necessary precision, the level of uncertainties in the application safety data package which would trigger the requirement for the 90-day studies on a case by case basis.” [13] GM crop whole food/feed testing therefore continues to be a mandatory element of the EU risk assessment process.

Since the crop under evaluation in GM crop testing scenarios is specifically intended as a food/feed component, some aspects are conceptually different compared to testing chemical compounds (applied as food or feed additives in low doses), and thus need careful consideration [14]. One such consideration relates to the appropriate dose level to evaluate a GM crop as a component of an experimental diet. According to the EFSA guidance for risk assessment of food and feed from genetically modified plants, “the highest dose level should not cause nutritional imbalance or metabolic disturbances in the test animal” [14]. Evidently, excessive dosing of any given feed component, irrespective of its GM properties, can interfere with the safety evaluation and can lead to the misinterpretation of observed effects. The proper feed component level to obtain nutritionally balanced diets should therefore be carefully established for any specific crop species/animal test-species combination, for example based on a review of literature and/or preliminary studies.

Another important challenge for the safety evaluation of GM food components relates to data interpretation relative to reference or control diets. On the one hand, GM risk assessment generally involves a comparative approach, where the GM crop is compared to its conventional wild type (WT) counterpart (i.e., the non-GM isogenic variety, or a genotype with a genetic background as close as possible to the GM plant) [6], known for its history of safe use [14]. On the other hand, any potential difference in biological effects between GM-containing and conventional feeds should be interpreted relative to the natural response variation of the analyzed endpoints (i.e., the range of biological values that is considered normal for a particular endpoint) to allow proper assessment of the toxicological relevance. The use of equivalence testing methods, a well-established statistical concept in the regulatory framework of novel drug testing, has recently been proposed for this purpose within the context of the evaluation of GM crop toxicity data obtained from animal feeding trials by van der Voet et al. [15]. The use of equivalence testing for assessing potential effects of GM crops is based on the general consensus that the difference between the GM crop and its conventional control should not necessarily be zero in the traditional statistical sense, but should be sufficiently small [15,16]. The calculation of threshold equivalence limits (ELs) is used as a method for quantifying the range within which a GM crop and its control are considered equivalent. One approach for setting such ELs is the specification of fixed values (e.g., as deemed appropriate by regulators). Another approach is the use of the biological variation of endpoint responses after feeding with various non-GM varieties as a reference dataset to generate ELs. Information on this natural response variation can be obtained from either historical data, or from feeding trials using commercially available non-GM reference varieties of the same crop species as the GM component under evaluation [6].

Fish were introduced as test animals in the framework of GM crop risk assessment to investigate the safety of these crops for aquaculture purposes (i.e., to be used as a fish feed ingredient) [17]. The most popular fish species that have been used for these purposes are Atlantic salmon [18,19,20,21,22,23,24,25] and rainbow trout [26,27]. Zebrafish have been used as a model for nutritional research and human metabolic diseases because of their considerable genetic identity and key metabolic similarities with humans [28,29,30], and the zebrafish has recently been proposed as a model for the safety evaluation of GM crops [31,32,33]. An important consideration in this respect is that the amount of crop material (non-GM and GM) required to perform the feeding trials is smaller compared to rodent trials, which may be relevant in cases where available materials are limited. The development and optimization of zebrafish whole food/feed testing methods, complementary to the existing rodent feeding trial, would therefore be a valuable addition to the GM safety assessment toolbox to aid risk assessors in decision-making processes. 

The aim of the present study was to develop and optimize methods that are required for advancing the use of zebrafish feeding trials for the toxicological safety evaluation of GM crops. We used maize as a crop species to design a case study. Numerous GM maize crops have been developed and cultivated, and it is one of the most important GM crop species worldwide in terms of production volume and acreage [34]. In the EU, 25 GM maize events have been authorized for food and feed purposes (EU register of authorized GMOs, January 2019), out of 62 registered events in total. In summary, our results demonstrate the need for preliminary feed tolerance testing to assess potential effects of feed component-related properties on the overall nutritional balance. Further, natural response variation datasets for various zebrafish endpoints were established when feeding with different non-GM maize cultivars. The natural response variation data were used in equivalence testing methods to calculate threshold equivalence limits as a method for quantifying the range within which a GM crop and its control are considered equivalent.

## 2. Results

### 2.1. Evaluating the Maize Substitution Level

#### 2.1.1. Diet Composition

The macronutrient composition of the different experimental diets was analyzed and gross energy values are given in Table 1. The coefficient of variation (CV) was determined for each macronutrient and for the calculated gross energy values of the experimental diets. A good feed intake was visually confirmed for all diet groups.

#### 2.1.2. Biological Parameters

##### Maize Substitution Range Finding

**Figure 1:**
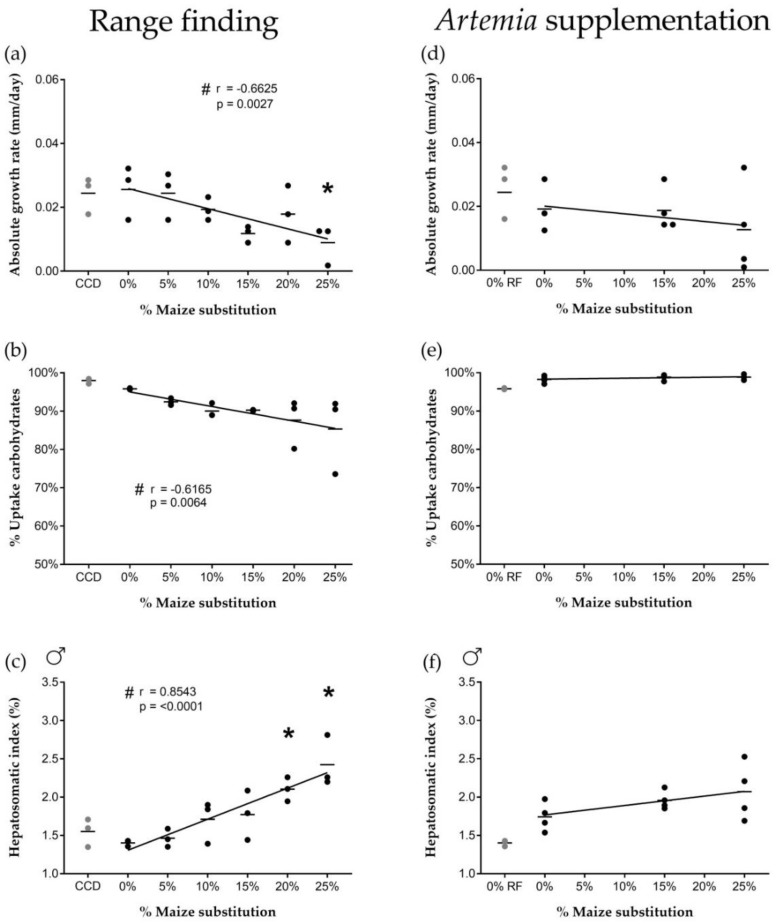
Effects of increasing dietary maize substitution levels. Range finding feeding trial: Increasing maize substitution levels resulted in (**a**) a significant decrease in the absolute growth rate (AGR) (*n* = 3), (**b**) a significant decrease in the % uptake of carbohydrates from the feed (*n* = 3) and (**c**) a significant increase in the hepatosomatic index (HSI) of male fish (*n* = 3). *Artemia* supplementation feeding trial: After the introduction of *Artemia* in the diets, increasing maize substitution levels no longer significantly affected (**d**) the AGR (*n* = 4), (**e**) the % uptake of carbohydrates from the feed (*n* = 4) or (**f**) the HSI of male fish (*n* = 4). Circles represent biological replicates; horizontal lines represent the mean of all replicates; *: significantly different from 0% maize (*p* < 0.05); #: significant correlation between maize substitution level and the respective parameter (*p* < 0.05); CCD: commercial control diet; 0% RF: 0% maize substitution values derived from the range finding feeding trial.

Maize substitution levels did not affect feed intake. All experimental diets were ingested within 5 min after feeding (i.e., 1.25% of the average initial body weight per feeding) and no excess feed was observed. No significant differences were observed between the commercial control diet (CCD) group and 0% of maize substitution (i.e., 25% of wheat) for any of the biological evaluated parameters. The relative condition factor (RCF) values were comparable for all different diet groups at all time points (day 0, 14 and 28) indicating a good general health condition of the fish during the experiment (Table 1). However, increasing the maize substitution level in the diet significantly decreased the absolute growth rate (AGR) of the zebrafish (Figure 1a). Furthermore, fish fed at a 25% maize substitution level showed a significantly lower growth rate compared to fish fed at 0% maize substitution.

**Figure 2:**
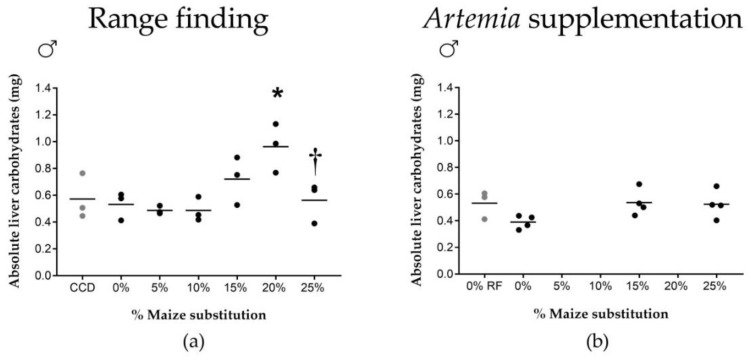
Effects of increasing dietary maize substitution level on liver carbohydrate content. (**a**) Range finding feeding trial: Increasing maize substitution levels resulted in an increase in the absolute amount of carbohydrates per pooled liver sample in male fish (*n* = 3) between 0 and 20% maize substitution; (**b**) *Artemia* supplementation feeding trial: No differences were observed in the absolute amount of carbohydrates in livers of male fish for the evaluated maize substitution levels (*n* = 4). *: significantly different from 0% maize (*p* < 0.05); †: significantly different from 20% maize (*p* < 0.05); CCD: commercial control diet; 0% RF: 0% maize substitution values derived from the range finding feeding trial.

Increasing dietary maize substitution levels resulted in a significant decrease in carbohydrate uptake from the feed (Figure 1b). No significant effects were observed when comparing the different maize substitution percentages to 0% maize. Also, no effects were observed on the lipid/protein uptake (Figure S1.1). Increasing maize substitution levels resulted in increased male hepatosomatic index (HSI, Figure 1c). The HSI of males fed with either 20% or 25% maize substitution was significantly higher compared to 0% maize. The level of maize substitution did not affect the HSI in female fish (Figure S1.2). No significant differences were found for the concentrations of energy reserves in liver or muscle for either sex (Figures S1.3–1.6). In absolute values, the total amount of carbohydrate per pooled liver sample significantly increased between 0 and 20% maize substitution in male fish (Figure 2a), but dropped again to control levels at 25% maize substitution. This pattern was not observed in female fish, nor for the other energy components (Figures S1.7–1.8).

##### *Artemia* Supplementation Feeding Trial

Maize substitution levels did not affect feed intake (no excess feed was observed) and no significant differences were observed in RCF of the fish (Table 1). Figure 1 shows that after supplementation of *Artemia*, maize substitution no longer resulted in decreased growth rate or increased male HSI (Figure 1d,f). Moreover, the decreased carbohydrate uptake from the feed observed during the range finding experiment was no longer observed (Figure 1e). The lipid/protein uptake (Figure S1.1), the female HSI (Figure S1.2) and the concentrations of energy reserves in liver and lateral muscle tissue (Figures S1.3–1.6) remained unaffected by varying maize substitution levels. Finally, the differences in the absolute amount of carbohydrates in male livers during the range finding experiment could not be directly compared to the male liver carbohydrates measured during the *Artemia* supplementation trial since 20% maize substitution was not included in the experimental design (Figure 2b). Whether the absolute amount of carbohydrates in male livers was no longer affected after supplementation of *Artemia* could therefore not be determined. No differences were observed in female fish, nor for the other energy components (Figures S1.7–1.8).

#### 2.1.3. Microarray Analysis

**Figure 3:**
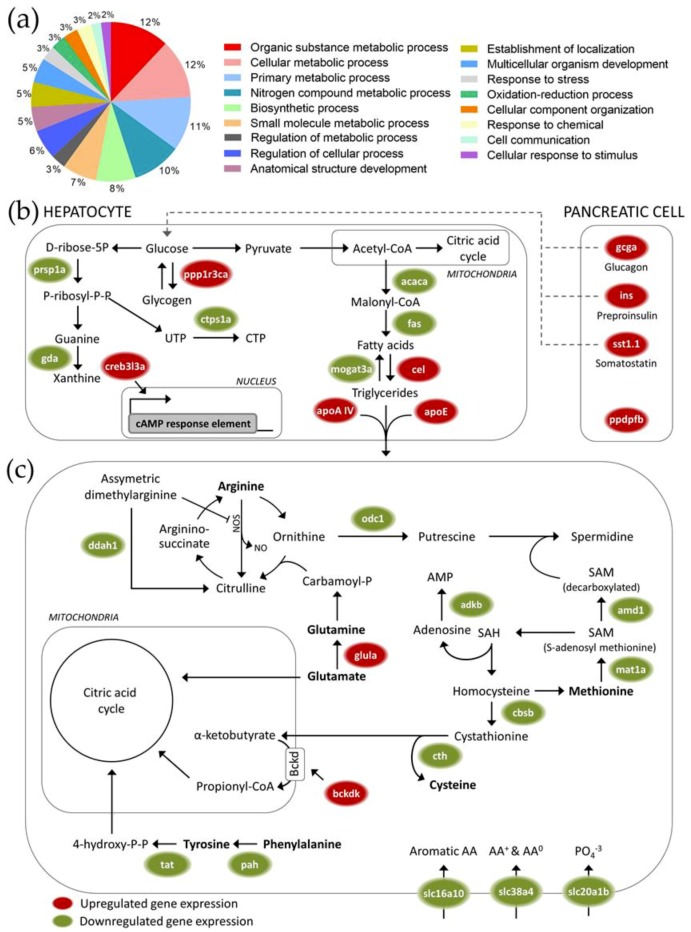
Transcriptional effects in zebrafish liver after feeding with 25% of maize substitution. (**a**) Pie chart summarizing the GO classes affected by feeding with 25% maize. Most differentially transcribed genes (63%) were related to metabolic processes. Details are given for affected pathways related to (**b**) carbohydrate, lipid and purine/pyrimidine metabolism and (**c**) amino acid metabolism. Green indicates downregulation and red indicates upregulation relative to 0% maize substitution (false discovery rate: *p* < 0.05). Dotted arrows represent endocrine regulation, solid arrows indicate pathway conversion steps.

Liver mRNA transcript levels were studied using microarray analysis after feeding with *Artemia*-supplemented diets (Gene Expression Omnibus: Series GSE121185, https://www.ncbi.nlm.nih.gov/geo/query/acc.cgi?acc=GSE121185). When comparing the 25% to the 0% maize diet independent of sex, 115 differentially expressed transcripts (false discovery rate: p < 0.05) were identified (Table S2.1), with 72 that were upregulated in response to increasing maize substitution levels, and 43 that were downregulated. The 107 transcripts with a known annotation were associated with 17 different gene ontology (GO) classes (Figure 3a), which were primarily associated with metabolic processes (63% of all transcripts). 

Figure 3b depicts the differentially expressed transcripts associated with carbohydrate, lipid and purine/pyrimidine metabolic pathways. The transcription of three key islet cell pancreatic hormones (glucagon, preproinsulin and somatostatin), and of the pancreatic cell differentiation and proliferation factor was upregulated after feeding with 25% of maize substitution. Further, transcripts related to carbohydrate metabolism were differentially expressed and transcripts coding for enzymes involved in the de novo lipogenesis were downregulated. The transcriptional expression of a lypolytic enzyme and two different apolipoproteins was upregulated. Transcription of enzymes involved in the synthesis of nucleotides (purines and pyrimidines) was downregulated, as were many genes associated with amino acid metabolism (Figure 3c). Transcription of three different solute carriers was also downregulated. A heat map of the transcriptional patterns described in Figure 3 is provided as Figure S2.1. The remaining 55 unique affected transcripts are given in Figure S2.2. Most of these genes were involved in growth, transport, oxidative stress, immunological, translational and transcriptional processes.

### 2.2. Estimating the Natural Response Variation

The macronutrient composition of the 10 experimental diets, each containing 15% of a different maize variety (MV), is given in Table 2. The variation in macronutrient composition as estimated by the coefficient of variation was higher compared to the experimental diets used during the range finding trial (Table 1), where only one maize variety (MV6) was used in different percentages. For example, the diet containing maize variety 9 (MV9; see Table S3.1), a sweet maize variety, had a high carbohydrate and lipid content while the diet containing the silo maize variety 8 (MV8) showed a rather low carbohydrate content. 

The natural response variation of the various biological endpoints (see Table S4.1 for data) was estimated by recording zebrafish responses to feeding with the 10 different non-GM maize reference varieties and used for evaluating the use of equivalence testing.

### 2.3. Evaluating the Use of Equivalence Testing

**Figure 4:**
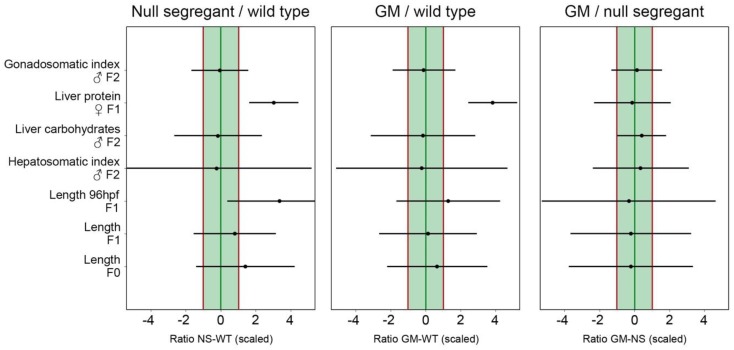
Equivalence testing based on the distribution-wise equivalence (DWE) criterion. Equivalence (DWE) tested for the contrasts GM vs. null segregant (NS), GM vs. wild type (WT) and NS vs. WT for a selection of endpoints: length, hepatosomatic index, liver carbohydrate and protein contents, gonadosomatic index. Mean equivalence limit scaled differences are presented as a black dot, with a 95% confidence interval. The vertical red lines represent the equivalence limits (ELs) (–1,+1), calculated based on the non-GM reference variation dataset.

For the selected endpoints, traditional statistical testing methods were applied first (see Table S4.2 for data). Figure 4 provides a few examples illustrating the concept of equivalence testing. The estimated variation used for calculating equivalence between the different diet groups are provided in Table S5.2. Differences between the diet groups as calculated using the distribution-wise equivalence (DWE) criterion were scaled based on the natural response variation.

The lowest likelihood of equivalence was found for the null segregant (NS)–wild type (WT) contrast, for which equivalence was not likely for three endpoints since the central point estimates for adult and larval length, as well as liver protein content, were outside the equivalence limit range. For the GM–WT comparison, the central point estimates of two endpoints (larval length and liver protein content) were outside the equivalence limit range. Interestingly, the NS–WT controls and GM–WT diet groups seem less equivalent than GM and NS, for which equivalence was ‘more likely than not’ for all endpoints, as all central point estimates were within the equivalence limit range.

## 3. Discussion

### 3.1. Physiological Effects of Maize Substitution and Diet Composition

Isoenergetic dietary substitution of wheat by maize affected carbohydrate uptake from the feed, which was associated with altered biological processes such as growth. Increased maize substitution resulted in a decreased absolute growth rate of the fish, a decreased carbohydrate uptake from the feed and an increased hepatosomatic index of male fish. Wheat and maize have similar carbohydrate contents (±70% starch), and the carbohydrate content of the different diets was similar (31.1 ± 2.4 g/100 g). The observed effects were therefore most likely related to nutrient digestibility and processing differences between maize and wheat by zebrafish. Such differences are commonly associated with properties inherent to the botanical origin of cereal grains [35]. For example, wheat starch granules are smaller (22–25 μm) compared to maize starch granules (35–40 μm) [36], resulting in a higher surface to volume ratio and thus better accessibility for digestive enzymes. Despite fish species-specific differences in overall carbohydrate digestibility, a lower digestibility of maize compared to wheat has been reported for gilthead sea bream [36,37], European sea bass juveniles [38], Juniá catfish and Nile tilapia [39].

Lower carbohydrate digestibility in maize-rich diets could have contributed to decreased growth rates. The addition of carbohydrates to the diet administered as wheat has been shown previously to positively influence zebrafish growth [40], and better growth performance of fish fed with wheat as opposed to maize has been demonstrated in gilthead sea bream [36,37]. While our carbohydrate uptake and growth rate data were derived from pooled male and female fish, we have shown an increase in liver carbohydrate stores at a 20% maize substitution level in males specifically, associated with a significant increase in male HSI. Females did not show an increased HSI or altered glycogen storage capacity, suggesting sex-specific differences in carbohydrate digestibility and storage.

Effects of high maize levels in feed on carbohydrate processing were no longer observed after supplementation of newly-hatched *Artemia* nauplii to the experimental diets. This live feed is characterized by a balanced nutritional profile considered ideal for zebrafish [41,42], and thus appears to have compensated for a decrease in available dietary carbohydrates in maize-rich diets (e.g., by providing a net energy surplus allowing fish to allocate more energy to biological processes such as growth).

### 3.2. Hepatopancreatic Transcriptome Responses to Maize Substitution

As the pancreatic tissue in zebrafish is closely intertwined with the liver, the tissue samples that were analyzed consist of both hepatocytes and pancreatic cells. The hepatopancreatic transcriptome analysis was designed to detect effects of high maize levels in feed that are subtler than the apical effects on growth and hepatosomatic index described earlier, but could still be important in interpreting potential effects of the presence of GM crops in feed in cases where physiologically equivalent control and GM crop-supplemented diets are used. All fish were therefore given balanced *Artemia*-supplemented diets for this analysis. 

Carbohydrate metabolism was one of the most clearly affected processes at the level of gene transcription after feeding at a high (25%) maize substitution level. The upregulated transcription of genes involved in insulin metabolism-related processes [43,44], hepatic glucose production through the transcriptional activation of key regulators of the gluconeogenesis [45], and inhibition of glycogen synthesis after feeding at high maize substitution levels suggest a potential compensation for the lower carbohydrate digestibility of maize-rich diets. Next to genes that are directly involved in carbohydrate metabolism, multiple genes involved in fat metabolism were found to be affected as well. Upregulated hepatic lipolysis and lipid transport/export protein transcripts suggest an increased dependency on fatty acids as the primary fuel in both liver and the rest of the organism, as well as an increased production of glycerol as a substrate for gluconeogenesis. On the other hand, high maize levels in feed triggered downregulation of many transcripts associated with amino acid metabolism. Overall, our data thus suggest a shift towards increased gluconeogenesis, glycogenolysis and fatty acid catabolism in response to the possible reduction of available glucose that is associated with maize-rich diets, but not to the extent where protein and amino acid catabolism is activated for producing alternative fuels.

Next to the altered energy metabolism pathways, genes associated with response to stress were differentially expressed after feeding at a high maize substitution level. Overall, an increased stress response could have contributed to the physiological effects during the range finding feeding trial. A transcriptional upregulation of genes involved in oxidative stress-related processes was observed, which could be an early sign of cytotoxicity. Feeding at high maize substitution levels could therefore have induced hepatotoxicity, which could be related to the significantly increased (male) hepatosomatic index. However, the dietary supplementation of *Artemia* seemed to minimize potential stress-induced responses at the physiological level.

### 3.3. Using Natural Response Variation Datasets for Equivalence Testing

The use of equivalence testing methods has been proposed to statistically evaluate whether a potential difference between the GM crop and one of its controls exceeds the reference natural response variation, and should therefore be considered as toxicologically relevant [15,16,46,47]. Although the EFSA does currently explicitly recommend the use of equivalence testing as a statistical approach for assessing the biochemical properties of the GM plant itself relative to its control and naturally related plants [46], it is important to note that equivalence testing methods have currently not yet been adopted in formal GM crop whole food/feed animal testing guidelines, and case studies are needed to evaluate their potential use [15]. To illustrate the application of equivalence testing methods using zebrafish data, we assessed a broad selection of endpoints at different levels of biological organization. The degree of natural response variation differed strongly between endpoints (see Table S4.1). Indeed, feeding with different non-GM maize cultivars caused high levels of variation for some zebrafish responses (e.g., energy reserves in liver tissue), but low levels of variation in other cases (e.g., hepatosomatic index).

In many cases, threshold limits for demonstrating equivalence are set using fixed values. For instance, the use of one standard deviation (SD) has been considered as an appropriate EL for GM crop safety evaluation [6] in cases where previous toxicity tests have shown that an effect size (i.e., the observed difference between dietary treatment groups) of one SD or less is of little toxicological relevance [47]. However, because some biological processes are known to be characterized by a higher response variation compared to others, equivalence limits cannot be extrapolated from one endpoint to another without additional, dedicated toxicity testing. In general, fixed ‘one-size-fits-all’ equivalence limits are therefore not necessarily representative of the actual natural response variation [48], and the use of additional data to generate threshold ELs based on endpoint-specific natural response variation, like we did using the DWE criterion [15], is likely to result in more accurate safety assessments. Within this context, the EFSA primarily advises the use of historical control data, compiled from earlier feeding trials using different non-GM control crops, for estimating the natural variation while reducing the number of animals required. However, historical data should only be used if generated by the same laboratory within 5 years preceding the study in question (OECD, TG452 [49]). In practice, and in particular for zebrafish studies for which such historical datasets are often not available at all, natural response reference variation datasets will therefore need to be generated in parallel to the actual GM feeding trials in most cases. 

Overall, the between-replicate variation of the data generated in the present study was large compared to other sources of variation (see Table S5.2), resulting in broad confidence intervals exceeding the EL range. Increasing the number of biological replicates therefore is a strategy that could be considered. Nonetheless, the central point estimates were within the EL range for the GM–null segregant comparison, meaning that equivalence was more likely than not for these two dietary treatments. The NS and WT control on the one hand, and the GM and WT control on the other hand, seemed less equivalent. In other words, the zebrafish responses to GM-containing feed were more similar to null segregant-containing feed than to WT control-containing feed. These findings suggest that the transformation process itself can be an important source of biological variation. The use of a null segregant control should therefore be considered in addition to the WT conventional counterpart as a valuable addition to standard procedures for GM safety assessment.

### 3.4. Recommendations and Conclusions

Within the context of the mandatory whole GM food/feed feeding trials that are part of the GM crop risk assessment process in European and other legislations, we aimed to develop and optimize methods for advancing the use of zebrafish feeding trials for the toxicological safety evaluation of GM crops using maize as a crop species in a case study. First, our results support the need for preliminary testing to assess potential feed component-related effects on overall nutritional balance. In our case, simply using a different carbohydrate source could sufficiently alter the nutritional balance of the diet to affect important physiological processes, despite a good general acceptance and equivalent macronutrient composition in the experimental diets. A separate step involving a feeding trial for the evaluation of component substitution levels could therefore be a valuable addition to zebrafish and other GM safety testing strategies. Next, an adequate substitution level for the actual GM component evaluation trial can be selected, which should be kept below nutritional and physiological effect limits, while still allowing the detection of potential effects of the GM component under assessment. Based on our results, we suggest a substitution level of 15% in GM maize feeding trials using zebrafish. Of course, the potential consequences of feed component substitution are largely dependent on the properties of the crop species under evaluation, and the selection of an adequate substitution level should therefore be made on a case by case basis. Specifically for zebrafish feeding trials, dietary supplementation with *Artemia* (or similar) could be considered as an additional measure to achieve balanced diets. Furthermore, commercial control diets (relevant for assessing feed formulation) and null segregant controls (relevant for assessing potential effects of the transformation process) are currently not included in GM risk assessment strategies, but would be valuable additions. To provide more detailed information on the mechanistic basis of observed biological responses, specific genes of interest could be selected for routine qPCR analysis. Finally, to facilitate the assessment of the toxicological/health relevance, a potential effect of a GM crop compared to its control identified using traditional statistical testing methods should ideally be interpreted relative to the natural response variation of the biological endpoint of concern. The natural response variation can be used as a reference dataset in equivalence testing methods to scale the observed differences between GM crop and control.

## 4. Materials and Methods 

### 4.1. Fish Husbandry and Ethics Statement

Adult zebrafish (*Danio rerio*) from an AB wild type line, maintained in Zebrafishlab at the University of Antwerp (Belgium) were used for all experiments. Fish husbandry was carried out in strict accordance with the EU Directive on the protection of animals used for scientific purposes (2010/63/EU). The presented work was approved by the Ethical Committee for Animals of the University of Antwerp (project number 2014-28). Reconstituted freshwater was used as fish medium and was prepared from reverse osmosis water (Werner, Leverkusen, Germany) by adjusting the conductivity to 500 ± 15 µS/cm (total hardness 45 mg/L CaCO_3_) using Instant Ocean Sea Salt (Blacksburg, VA, USA) at pH 7.5 ± 0.3 (adjusted using NaHCO_3_). Fish were kept at a density of 20 animals per 3.5 L tank in a ZebTEC stand-alone system (total water volume 250 L; Tecniplast, Buguggiate, Italy) at 28 °C ± 0.2, a 14/10 h light/dark cycle and continuous biological filtration and water recirculation. Ammonium, nitrite and nitrate levels of the system were monitored twice a week using Tetratest kits (Tetra, Melle, Germany), and values were always below 0.25, 0.3 and 12.5 mg/L, respectively. Before initiating a feeding trial, adult zebrafish were kept under these standard conditions for at least one month while being fed a commercial control diet (Biogran Medium).

### 4.2. Experimental Diet Formulation

Experimental diets (Table 3) were formulated based on the nutrient composition of a typical commercial fish feed and produced at the Laboratory for Feed Technology at Ghent University. The production protocol is available in Supplementary Materials S6. All maize crops were cultivated at the Institute for Agricultural and Fisheries Research (ILVO; see Table S3.1). All ingredients were analyzed for pesticide residues (Primoris CVBA, Zwijnaarde, Belgium) and mycotoxins (Centre of Excellence in Mycotoxicology and Public Health, Ghent University; see Supplementary Materials S3) [50]. No pesticide residues were found, and in cases where mycotoxins were detected, all values were within the known guidance values for animal feed (Table S3.1).

For evaluating the maize substitution level (see Section 4.3.1), experimental diets were developed to be isoenergetic by gradually substituting wheat (a cereal grain source similar to maize) with maize, resulting in six experimental diets ranging from 0 to 25% of maize (corresponding to 25 to 0% of wheat, respectively; see Table 3). For estimating the natural response variation (see Section 4.3.2), fish were fed with 10 different maize reference varieties while keeping the substitution level fixed at 15% (marked with an asterisk in Table 3). The reference varieties were selected to include a large variation in characteristics (e.g., carbohydrate content) to achieve maximal variation in zebrafish responses (Table S3.1). The same feed formulation and maize percentage (15%, marked with asterisk in Table 3) were used during the transgenerational experiment for evaluating the use of equivalence testing, resulting in three experimental feeds containing either GM maize, WT maize or NS maize (see Section 4.3.3).

### 4.3. Overall Experimental Design

We first investigated the effects of maize substitution in the diets of zebrafish using non-GM maize. Two feeding trials were carried out for that purpose. First, the maximum level of maize substitution tolerable for zebrafish was determined. In a second feeding trial, two questions were addressed: (1) does the addition of live *Artemia salina* nauplii to the experimental diet compensate for adverse effects observed at high maize substitution levels, and (2) does the presence of maize in the diet affect mRNA transcriptional profiles in the zebrafish liver.

After having established the optimal GM substitution level and general feeding conditions, the natural variation of the different zebrafish endpoints was determined after feeding with 10 non-GM maize reference varieties.

Finally, the use of equivalence testing in zebrafish-based GM feeding trials was evaluated. To provide a number of relevant examples of equivalence testing datasets, we used data on an experimental GM maize and two different controls (wild type and null segregant) that originated from a transgenerational feeding trial carried out in our laboratory. The transgenerational study aspects themselves, however, are not the focus of the present study.

#### 4.3.1. Evaluating the Maize Substitution Level

First, a range finding experiment was carried out using six experimental diets (0, 5, 10, 15, 20 and 25% of maize substitution, see Section 4.2 for feed formulation) and one reference commercial control diet (Biogran Medium, Prodac International, Cittadella, Italy). Each diet group consisted of three biological replicates (10 male and 10 female fish per replicate). Fish were fed twice a day (at 9 a.m. and 3 p.m., 1.25% of the average wet weight at day 0 per feeding—i.e., 2.5% of the average initial wet weight per day in total) for 4 weeks. Relative condition factor (RCF, see Section 4.4) and length were determined at day 0, 14 and 28 (end) of the feeding trial to evaluate the general well-being of the fish (*n* = 60). Total fecal matter produced over 24 hours (i.e., one day of feeding) per replicate tank (*n* = 3) was collected during the final week of the feeding trial. At day 28, fish were not given any feed to facilitate dissection of the tissues and to avoid fecal contamination of the samples. Before dissection, fish were euthanized by an overdose of 300 mg/L tricaine methanesulfonate (MS222; pH 7.5; Sigma-Aldrich, St. Louis, MO, USA) followed by decapitation. Liver and lateral muscle tissue were immediately removed and pooled per replicate per sex (*n* = 3 per sex) for measuring energy reserves (see Section 4.5). Lateral muscle tissue was removed from the same area for all fish. Livers were weighed for calculating the hepatosomatic index (HSI). Tissues were frozen in liquid nitrogen and stored at –80 °C.

In a second feeding trial, three experimental diets were tested (i.e., 0%, 15% and 25% of maize substitution) representing a minimum, intermediate and high dietary maize level. Each diet group consisted of four biological replicates (10 males and 10 females per replicate). Fish were fed twice daily with experimental diets (2.5% average wet weight per day, 9 a.m. and 3 p.m.), and once daily with newly hatched *Artemia* nauplii (cysts purchased from Fleuren & Nooijen BV, The Netherlands; approximately 0.5% average wet weight at 12 a.m.) for 4 weeks. RCF and total fecal matter were determined as described earlier. Liver and lateral muscle tissue were isolated and pooled per replicate per sex (*n* = 4 per sex). Once livers were weighed for calculating the HSI, pooled liver tissues were homogenized in liquid nitrogen using a mortar and pestle to avoid RNA degradation. One aliquot of the liver tissue was preserved in QIAzol Lysis Reagent at −80 °C until RNA extraction. Male and female liver tissue derived from fish fed with 0 and 25% maize substitution was used for microarray analysis. A second liver tissue aliquot and the collected lateral muscle tissue were stored in –80 °C for the analysis of the energy reserves (see Section 4.5).

#### 4.3.2. Estimating the Natural Response Variation

**Figure 5:**
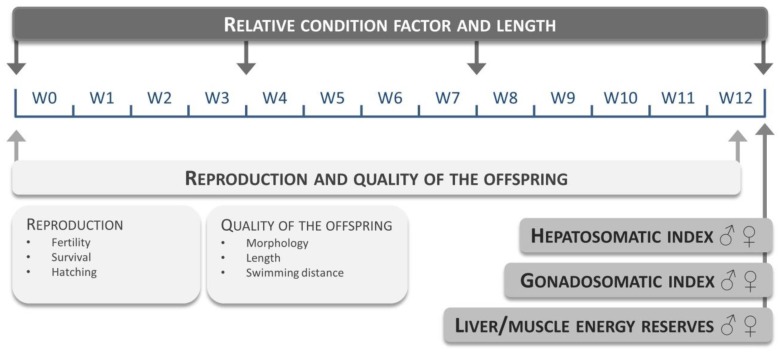
Estimating the natural response variation. Overview of the selected endpoints and corresponding time points measured during the natural response variation feeding trial. Relative condition factor and length were evaluated every 4 weeks, reproduction parameters and quality of the offspring were analyzed at the start of the experiment and at week 12. Hepatosomatic/gonadosomatic index and liver/muscle energy reserves were measured at the end of the feeding trial. W: week.

A total of 10 different non-GM maize reference varieties (see Table S3.1) were evaluated at a fixed maize substitution level of 15%. Each diet group consisted of three replicates (10 males and 10 females per replicate). Fish were fed three times a day: experimental feed at 9 a.m. and 3 p.m. (2.5% average wet weight per day) and *Artemia* nauplii at 12 a.m. (approximately 0.5% average wet weight) for 12 weeks. The evaluated endpoints and time points are given in Figure 5.

#### 4.3.3. Evaluating the Use of Equivalence Testing

For the purpose of evaluating the use of natural response variation data in an equivalence testing approach, a case study was developed by testing a non-commercial GM maize and two different types of controls. To provide a number of relevant examples of equivalence testing datasets, we used data that originated from a transgenerational feeding trial carried out in our laboratory, although the transgenerational study aspects themselves are not the focus of the present study. The GM maize had been developed previously and independently of the present study, and is characterized by an increased production of GA20-OXIDASE 1, an enzyme involved in the synthesis of gibberellic acid regulating growth and stretching of plant cells [51,52]. Controls included the wild type maize (WT; i.e., the original starting material for producing the GM maize), and the null segregant (NS; i.e., progeny plant having experienced the transformation process but lacking the transgene). Including these two controls allowed for the evaluation of potential effects of the genetic modification itself (GM maize versus NS), potential effects of the transformation process (NS versus WT) and potential effects of both aspects combined (GM maize versus WT). More details on the experimental design of the transgenerational feeding trial, including raising the different generations, is provided in the Supplementary Materials S7.

### 4.4. Relative Condition Factor and Tissue Somatic Indices

All fish were weighed (up to 0.01 g accuracy) and fork length was determined (up to 1 mm accuracy). The relative condition factor (RCF) was calculated using the body wet weight (W in g) and fork length (L in mm) [53]. Parameters a and b were derived from the weight–length relationship of all individual fish at the start of every feeding trial, described by: W = aL^b^ [54]. The RCF was calculated as RCF = W/aL^b^. The absolute growth rate (AGR) was calculated as AGR = (L_f_-L_i_)/t, with L_i_ as the mean initial fork length per replicate measured at the first day of the feeding trial; L_f_ as the mean final length per replicate; and t as time in days [55]. The hepatosomatic (HSI) and gonadosomatic indices (GSI; i.e., the weight of the liver and gonads respective to the total body weight of the fish) were calculated at the end of each feeding trial. Values were calculated based on pooled samples of 10 liver or gonadal tissues per sex per replicate, and HSI and GSI were calculated using the sum of the total body weight of the 10 corresponding fish.

### 4.5. Tissue Energy Reserves, Macronutrient Composition of the Diets and Feed Digestibility

After weighing, all tissue samples were homogenized in Milli-Q water using a TissueRuptor (Qiagen, Hilden, Germany). One aliquot (200 µL, approximately 8 mg tissue) of liver/muscle tissue homogenate was treated with 0.2 N perchloric acid to precipitate proteins. The supernatant was aspirated and transferred to a new recipient. Pellets were dissolved and total protein content was analyzed using the Bradford method (Bio-Rad Laboratories, Nazareth, Belgium), measuring spectrophotometric absorbance at 595 nm in a microplate reader (Synergy Mx, Biotek Instruments Inc., VT, USA). Bovine serum albumin (Merck, Belgium) in Milli-Q water was used as a reference curve to calculate protein concentrations. To determine the digestible carbohydrate content, the supernatant was incubated with Anthrone reagent and absorption was measured at 630 nm [56]. A standard curve was constructed using purified bovine liver glycogen (Merck, Belgium) in 0.2 N perchloric acid. A second aliquot was used for total lipid analysis. Total lipid extraction [57] was followed by an incubation period with 99.99% sulphuric acid. A calibration curve of tripalmitin (Acros Organics, Thermofisher, Geel, Belgium) in chloroform was used for calculating total lipid content measuring spectrophotometric absorption at 375 nm. Protocol details on measuring energy reserves are provided in Supplementary Materials S8.

The macronutrient composition of the diets was determined using the same methods as for the tissue samples. A portion of each diet was homogenized in Milli-Q water and an aliquot of 200 µL (2 mg) was used for analysis. The main digestible carbohydrate molecule in the diets is starch (theoretically 91% according to the diet formulation); a standard curve using maize starch in 0.2 N perchloric acid was therefore constructed. Gross energy values were calculated using gross energy contents of 17.5 kJ/g for carbohydrates, 24 kJ/g for proteins and 39.5 kJ/g for lipids [58]. To estimate the digestibility of the experimental diets, the composition of the fecal samples (measured in 200-µL aliquots containing 4 mg) was determined using the same methods. Digestibility/daily nutrient uptake from the feed was calculated by determining the ratio of the quantity of each component (i.e., carbohydrates, proteins and lipids) measured in the fecal sample per replicate relative to the quantity of that component present in the feed dosage of one day per replicate.

### 4.6. RNA Isolation, Labeling and Two-Color Microarray Analysis

The effects of feeding at 0, 15 and 25% maize substitution levels at the transcriptome level (see Section 4.3.1) were measured in liver tissue of both male and female fish using microarray analysis. RNA extraction was performed using QIAzol Lysis Reagent (Qiagen, Hilden, Germany), chloroform and 2-propanol according to the manufacturer’s instructions, and further purified using lithium chloride (see Supplementary Materials S2 for RNA purity and integrity data). RNA was reverse transcribed into cDNA, after which cRNA was constructed in the presence of Cyanine-3-CTP or Cyanine-5-CTP using the Low Input QuickAmp Labeling Kit Two-Color (Agilent Technologies, Diegem, Belgium) according to the manufacturer’s protocol. The labeled cRNA was purified using RNeasy Mini Kit (Qiagen, Hilden, Germany). Hybridizations were performed on Agilent zebrafish (V3) gene expression 4×44K microarrays (AMADID 026437, Agilent Technologies). A simple n-loop design [59] was used in which each sample was labeled once in Cy3 and once in Cy5, resulting in 12 arrays for 24 samples (0 and 25% maize, 4 biological replicates per diet, analyzed per sex). Every microarray contained 825 ng of both Cy3- and Cy5-labeled cRNA. Microarrays were incubated at 65 °C for 17 h in a rotating hybridization chamber at 10 rpm, washed with Agilent wash buffers and immediately scanned using a Genepix Personal 4100A confocal scanner (Axon Instruments, Union City, CA, USA) at a resolution of 5 µm. The photomultiplier tube voltages (PMT) for separate channels were adjusted to obtain an overall red/green ratio of 1 ± 0.3. Images were processed using GenePix Pro 6.1 software (Axon Instruments, Union City, CA, USA) for spot identification and quantification of the fluorescent signal intensities. The statistical processing of raw microarray data was carried out using the R package Limma as described by Vergauwen et al. [60]. Details on the statistical microarray data processing steps are provided as Supplementary Materials S2 [61]. The total number of significantly differentially expressed genes was calculated for three different contrasts: female 25 vs. 0% maize, male 25 vs. 0% maize and 25 vs. 0% maize independent of sex. Heat maps (Figures S2.1 and S2.2) were produced using the MultiExperiment Viewer software version 4.9.0 (MeV, http://mev.tm4.org). The gene ontology (GO) classes covering the differentially expressed genes were identified using the GO blast tool AmiGO (http://amigo.geneontology.org/amigo). Affected pathways were identified using the KEGG (Kyoto Encyclopedia of Genes and Genomes) pathway database (http://www.genome.jp/kegg/pathway.html).

### 4.7. Reproductive Performance and Quality of the Offspring

Spawning was induced by a morning light stimulus in each replicate tank. Eggs were collected, rinsed using reconstituted freshwater and fertility rate was visually determined under a Leica S8APO stereomicroscope. Fertilized eggs were transferred to 48-well plates (1 plate per replicate), each well containing 1 embryo in 1 mL medium, and reared in an incubator at 28.5 °C at a 14/10 h light/dark cycle. Mortality and hatching were monitored at 8, 24, 48, 72 and 96 h post fertilization (hpf). The quality of the embryos was evaluated at 96 hpf by assessing embryonic development (morphology—e.g., swim bladder inflation, length and swimming distance). To determine length, embryos were photographed together with a calibrator and images were analyzed using the ImageJ software (available at http://rsbweb.nih.gov/ij/). Swimming distance was recorded during a 40 min light period using a Zebrabox 3.0 video tracking device (ViewPoint, Lyon, France) and calculated using the Zebralab software (version 3.20.5.104).

### 4.8. Data Analysis

#### 4.8.1. Evaluating the Maize Substitution Level

Datasets were tested using parametric one-way analysis of variance (ANOVA), using a Dunnett’s post hoc multiple comparisons test comparing each experimental diet to the experimental diet containing 0% maize substitution. In cases where data were obtained for individual fish within replicate groups (e.g., RCF), an additional replicate-effect should be nested within the main factor of interest. In this case, mixed one-way ANOVA models were used to include both the random (i.e., replicate) and the fixed factor (i.e., diet group). Pearson’s r correlation was used to test the relationship between the maize substitution level and the effects on a given endpoint. To test the overall quality of the experimental diet, 0% maize substitution values of all endpoints were compared to the commercial control diet using an unpaired t-test. Data were considered significantly different when p-values were <0.05. Statistical analyses were performed using GraphPad Prism version 7.00 and R Statistical Software (R Core Team, 2018 version 3.5.0, https://www.Rproject.org/).

#### 4.8.2. Evaluating the Use of Equivalence Testing

A number of endpoints that were evaluated during the transgenerational trial (see Section 4.3.3) were selected as a case study. The data were first analyzed using traditional statistical testing methods (ordinary or mixed one-way ANOVA). Subsequently, equivalence was tested for these endpoints. Equivalence testing requires prior specification of criteria. The different strategies for setting criteria relevant to GM crop safety evaluation are discussed in Supplementary Materials S5. We applied the distribution-wise equivalence (DWE) criterion according to van der Voet et al. [15], as it allows simultaneous consideration of variation among non-GM reference groups (different non-GM maize cultivars), inter-replicate variation and variation among feeding trials. Equivalence limits were calculated based on our reference dataset on natural variation in zebrafish responses. The central point estimates presented in the present study represent the mean equivalence-limit scaled (based on non-GM reference data) differences between the dietary treatments under comparison. Briefly, when central point estimates are within the equivalence limits interval (–1,+1), it is considered that ‘equivalence is more likely than not’. If not only the central point but also its corresponding confidence intervals are within the (–1,+1) limits, this is considered as ‘proof of equivalence’ [15]. It is important to note however that a failure to demonstrate equivalence is not a proof of non-equivalence. The statistical approach for equivalence testing is described in more detail in Supplementary Box S5.1.

## Figures and Tables

**Table 1:** Nutritional composition and calculated gross energy of the experimental diets (% of maize substitution) and the commercial control diet (CCD).

Commercial Control Diet		Experimental Diets (% Maize Substitution)						
		0%	5%	10%	15%	20%	25%	CV ^2^
Macronutrient composition (g/100 g)								
Dry matter	93.0	90.0	90.4	90.4	90.8	90.8	90.6	0.3%
Carbohydrates	46.4	32.1	34.7	31.3	31.5	29.5	27.5	7.8%
Proteins	33.4	30.4	34.8	34.4	36.8	34.9	33.9	6.2%
Lipids	3.7	5.0	5.5	4.0	5.8	5.0	6.1	14.2%
Gross energy (kJ/g)	17.6	14.9	16.6	15.3	16.6	15.5	15.4	4.5%
RCF ^1^ “Range finding”								
Day 0	1.00 ± 0.14	1.01 ± 0.13	1.04 ± 0.15	1.01 ± 0.13	0.97 ± 0.12	0.99 ± 0.14	0.99 ± 0.13	13.4%
Day 14	1.00 ± 0.13	1.02 ± 0.13	1.03 ± 0.13	1.06 ± 0.15	1.00 ± 0.13	0.98 ± 0.13	0.97 ± 0.12	14.4%
Day 28	0.95 ± 0.14	0.98 ± 0.13	1.01 ± 0.17	0.99 ± 0.15	0.96 ± 0.16	0.96 ± 0.12	0.96 ± 0.12	14.4%
RCF ^1^ “*Artemia* supplementation”								
Day 0		1.00 ± 0.11			1.00 ± 0.10		1.01 ± 0.11	10.5%
Day 14		1.04 ± 0.11			1.03 ± 0.11		1.03 ± 0.12	10.8%
Day 28		0.98 ± 0.12			1.00 ± 0.12		1.02 ± 0.12	12.0%

^1^ RCF: relative condition factor values for all diet groups. Presented RCF values are mean ± standard deviation measured at day 0, 14 and 28 of the “Range finding” (*n* = 60) and “*Artemia* supplementation” (*n* = 80) feeding trial. ^2^ CV: coefficient of variation (commercial control diet excluded).

**Table 2:** Nutritional composition of the experimental diets containing 10 different maize reference varieties (MV 1–10; 15% total maize substitution) and calculated gross energy values.

	MV1	MV2	MV3	MV4	MV5	MV6	MV7	MV8	MV9	MV10	CV ^1^
Macronutrient composition (g/100 g)											
Dry matter	94.0	93.9	93.9	93.8	93.9	93.9	93.9	93.8	93.8	93.9	0.1%
Carbohydrates	38.1	32.2	37.2	30.6	36.9	35.9	38.1	28.0	61.6	33.2	24.8%
Proteins	34.5	43.8	24.4	30.6	37.0	32.6	21.8	26.9	28.5	29.6	20.7%
Lipids	5.9	2.9	4.2	4.7	4.8	5.8	6.1	5.1	6.6	4.2	22.0%
Gross energy (kJ/g)	17.3	17.3	14.0	14.6	17.2	16.4	14.3	13.4	20.2	14.6	13.3%

^1^ CV: Coefficient of variation.

**Table 3:** Formulation of the experimental diets (g per 100 g dry matter).

Ingredients	0%	5%	10%	15%*	25%
Maize ^1^	-	5.00	10.00	15.00	25.00
Wheat ^2^	25.00	20.00	15.00	10.00	-
Peas ^2^	33.45				
Fish meal ^2^	10.00				
Fish oil ^2^	5.15				
Wheat gluten ^3^	18.85				
Hemoglobin powder ^4^	5.00				
Barox dry ^5^	0.05				
Pro-bind plus ^6^	0.50				
Premix AB1 Fulda ^7^	1.50				
Monocalcium phosphate ^8^	0.50				
Milli-Q water	25.00				

^1^ All maize crops were cultivated specifically for this study at the Institute for Agricultural and Fisheries Research (ILVO, Belgium). ^2^ Aqua-Bio, Joossen-Luyckx B.V., Belgium; ^3^ BENEO GmbH, Germany; ^4^ Actipro 95, Veos, Belgium; ^5^ Antioxidant mixture, Kemin Industries, Inc., USA; ^6^ Binding agent, Sonac, The Netherlands; ^7^ INVE Belgium N.V.; ^8^ Omya SA/NV, The Netherlands.

## Supplementary Materials

Supplementary materials can be found at https://www.mdpi.com/1422-0067/20/6/1472/s1.

## Abbreviations

AGR	Absolute growth rate
ANOVA	Analysis of variance
CCD	Commercial control diet
CV	Coefficient of variation
DWE	Distribution-wise equivalence
EFSA	European Food Safety Agency
EL	Equivalence limit
GM	Genetically modified
GO	Gene ontology
GSI	Gonadosomatic index
hpf	Hours post fertilization
HSI	Hepatosomatic index
ILVO	Institute for agricultural and fisheries research
KEGG	Kyoto encyclopedia of genes and genomes
L	Fork length
NS	Null segregant
MV	Maize variety
OECD	Organization for Economic Co-operation and Development
PMT	Photomultiplier tube
RCF	Relative condition factor
RF	Range finding feeding trial
SD	Standard deviation
W	Weight/Week
WT	Wild type
