# Supplementary material for: Optimizing the Use of Zebrafish Feeding Trials for the Safety Evaluation of Genetically Modified Crops
Source: Int J Mol Sci. 2019 Mar 23;20(6):1472. doi: 10.3390/ijms20061472 (PMC6471220; doi:10.3390/ijms20061472)
Supplement: Supplementary file 1 [file ijms-20-01472-s001.pdf]

Supplementary Materials

Optimizing the use of zebrafish feeding trials for the safety evaluation of genetically modified crops

Isabelle J. Gabriëls [1], Lucia Vergauwen [1,2], Marthe De Boevre [3], Stefan Van Dongen [4], Ronny Blust [2], Sarah De Saeger [3], Mia Eeckhout [5], Marc De Loose [6] and Dries Knapen [1,*]

[1] Zebrafishlab, Veterinary Physiology and Biochemistry, Department of Veterinary Sciences, University of Antwerp, Universiteitsplein 1, 2610 Wilrijk, Belgium; isabelle.gabriëls@uantwerpen.be (I.J.G.); lucia.vergauwen@uantwerpen.be (L.V.)

[2] Systemic Physiological and Ecotoxicological Research (SPHERE), Department of Biology, University of Antwerp, Groenenborgerlaan 171, 2020 Antwerpen, Belgium; ronny.blust@uantwerpen.be (R.B.)

[3] Centre of Excellence in Mycotoxicology and Public Health, Department of Bioanalysis, Ghent University, Ottergemsesteenweg 460, 9000 Gent, Belgium; marthe.deboevre@ugent.be (M.D.B.); sarah.desaeger@ugent.be (S.D.S.)

[4] Evolutionary Ecology Group, Department of Biology, University of Antwerp, Universiteitsplein 1, 2610 Wilrijk, Belgium; stefan.vandongen@uantwerpen.be (S.V.D.)

[5] Department of Food Technology, Food safety and Health, Faculty of Bioscience Engineering, Ghent University, Valentin Vaerwyckweg 1, 9000 Gent, Belgium; mia.eeckhout@ugent.be (M.E.)

[6] Technology and Food Sciences Unit, Institute for Agricultural and Fisheries Research (ILVO), Burg. Van Gansberghelaan 115, 9820 Merelbeke, Belgium; marc.deloose@ilvo.vlaanderen.be (M.D.L.)

* Correspondence: dries.knapen@uantwerpen.be (D.K.); Tel.: +32-3-265-27-24

Table of contents

S1. Supplemental data of the evaluation of the maize substitution level 3
S2. RNA purity and integrity and statistical processing steps of raw microarray data 10
S3. Mycotoxin analysis 13
S4. Supplemental data natural response variation/transgenerational feeding trial 15
S5. Discussion of the equivalence criteria and statistical approach of equivalence testing 17
S6. Feed production protocol 21
S7. Experimental design transgenerational feeding trial and raising the next generation 22
S8. Energy reserves protocol details 23

S1. Supplemental data of the evaluation of the maize substitution level

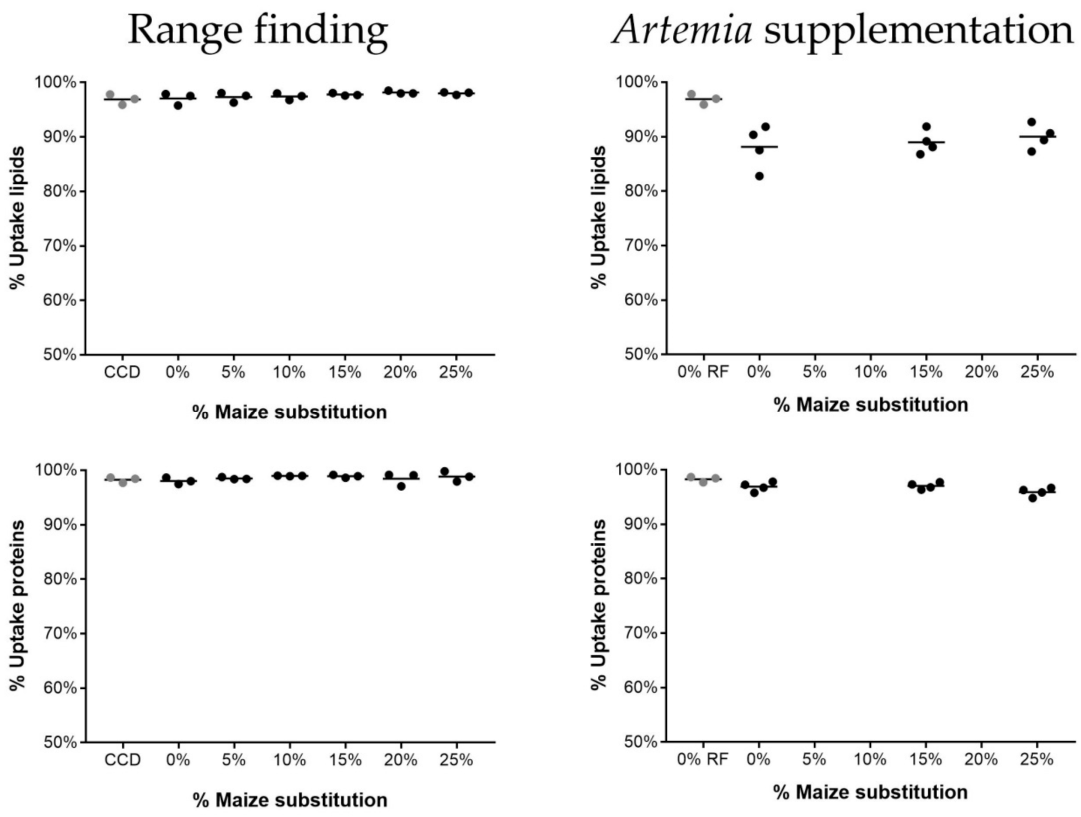

Figure S1.1: Uptake of lipids and proteins from the feed. The degree in dietary maize substitution did not affect the % uptake (Range finding: n = 3; *Artemia* supplementation: n = 4). Circles represent biological replicates; horizontal lines represent the mean of all replicates; CCD: Commercial control diet; 0% RF: 0% maize substitution values from the range finding feeding trial.

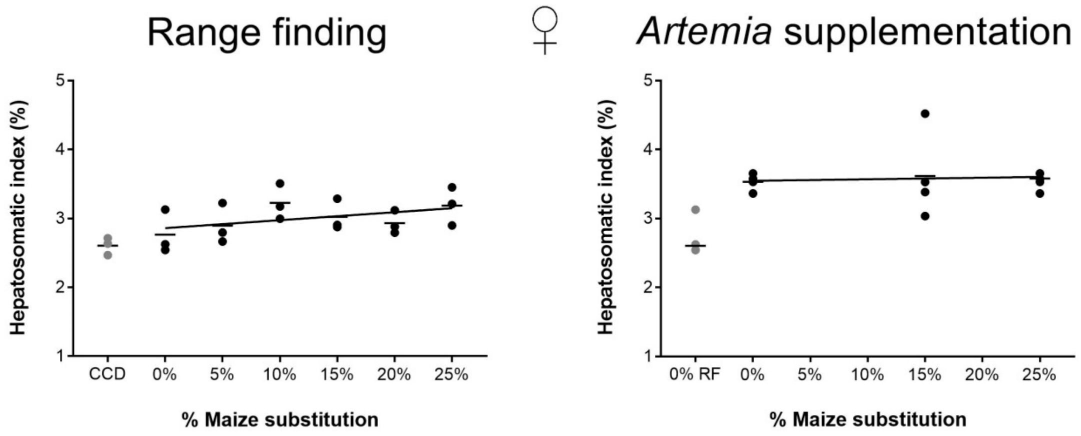

Figure S1.2: Hepatosomatic index of female fish. The degree in dietary maize substitution did not affect the hepatosomatic index of female fish (Range finding: n = 3; *Artemia* supplementation: n = 4). Circles represent biological replicates; horizontal lines represent the mean of all replicates; CCD: Commercial control diet; 0% RF: 0% maize substitution values from the range finding feeding trial.

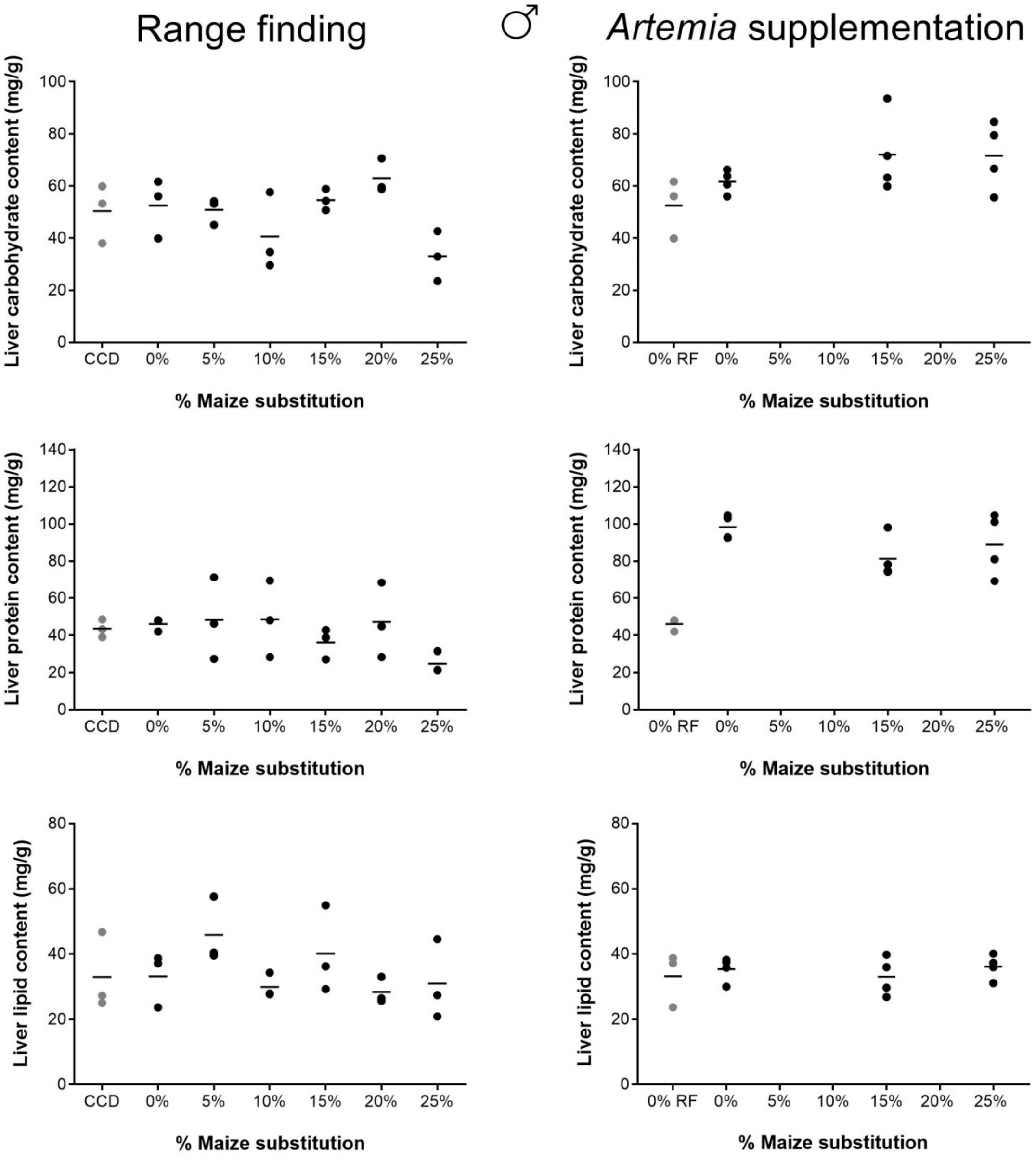

Figure S1.3: Concentrations of energy stores (from top to bottom: carbohydrate, protein and lipid content) in male liver tissue (Range finding: n = 3; *Artemia* supplementation: n = 4). Increasing the dietary maize substitution rate did not statistically affect energy reserves. Circles represent biological replicates; horizontal lines represent the mean of all replicates; CCD: Commercial control diet; 0% RF: 0% maize substitution values from the range finding feeding trial.

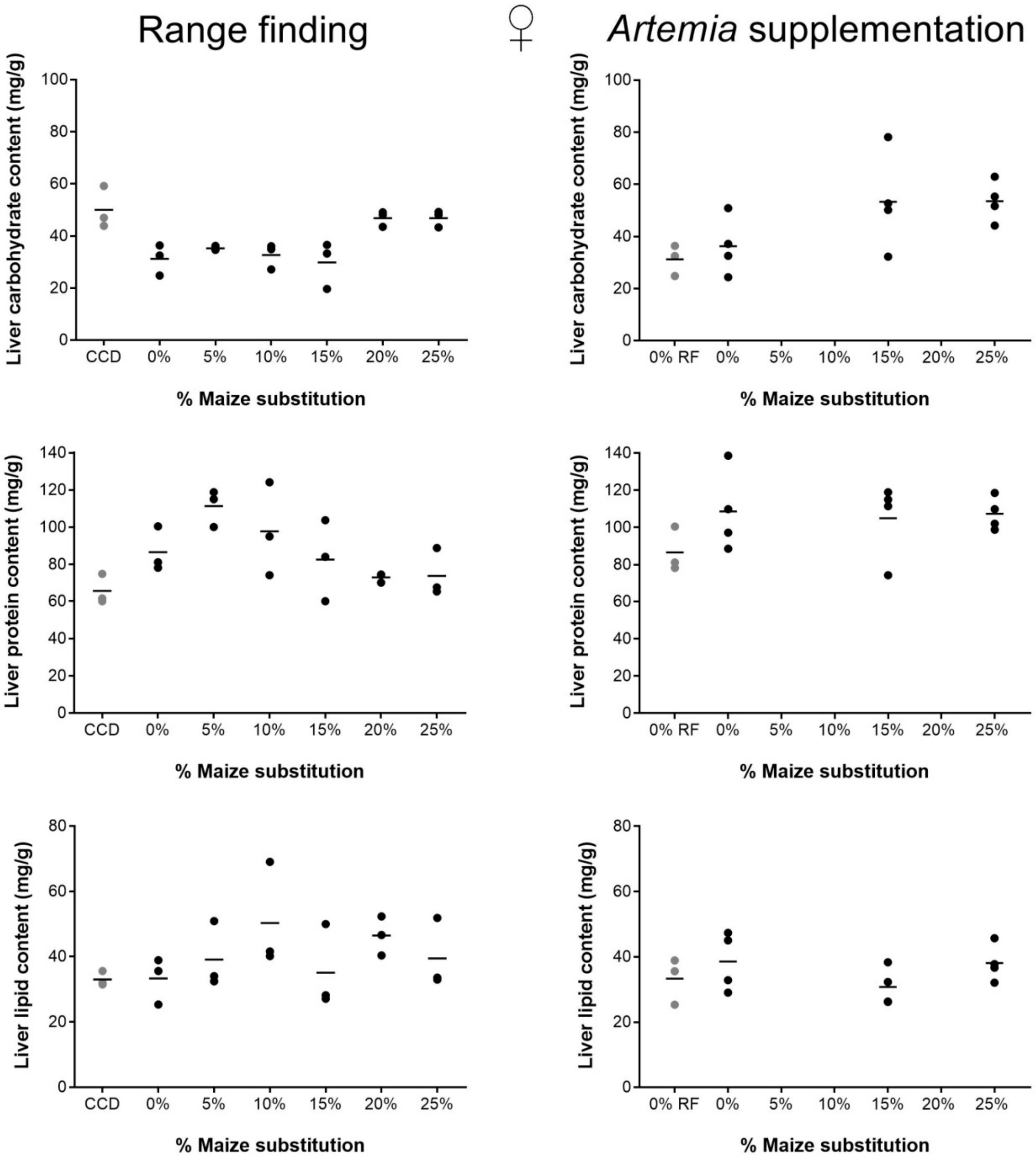

Figure S1.4: Concentrations of energy stores (from top to bottom: carbohydrate, protein and lipid content) in female liver tissue (Range finding: n = 3; *Artemia* supplementation: n = 4). Increasing the dietary maize substitution rate did not statistically affect energy reserves. Circles represent biological replicates; horizontal lines represent the mean of all replicates; CCD: Commercial control diet; 0% RF: 0% maize substitution values from the range finding feeding trial.

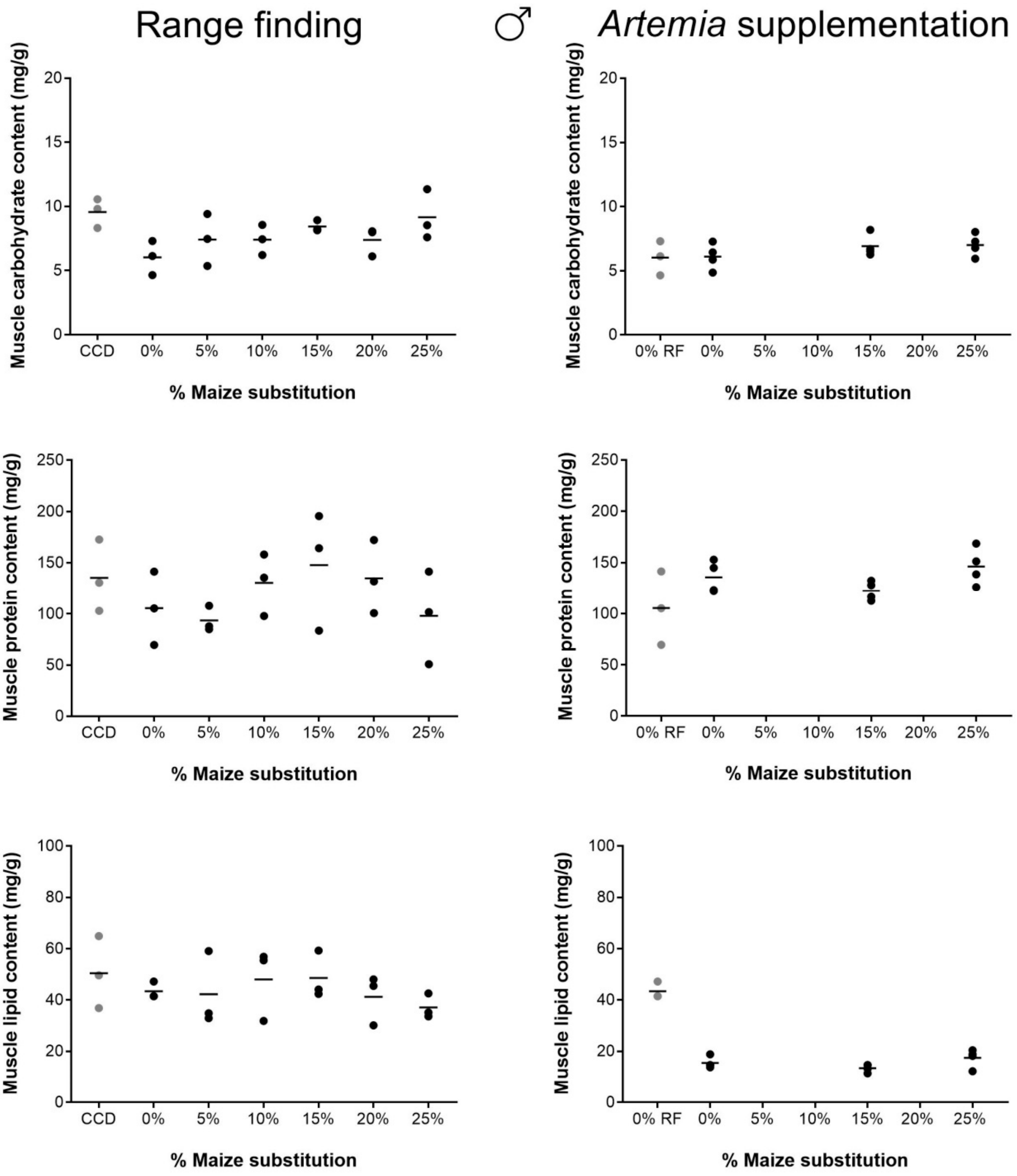

Figure S1.5: Concentrations of energy stores (from top to bottom: carbohydrate, protein and lipid content) in male muscle tissue (Range finding: n = 3; *Artemia* supplementation: n = 4). Increasing the dietary maize substitution rate did not statistically affect energy reserves. Circles represent biological replicates; horizontal lines represent the mean of all replicates; CCD: Commercial control diet; 0% RF: 0% maize substitution values from the range finding feeding trial.

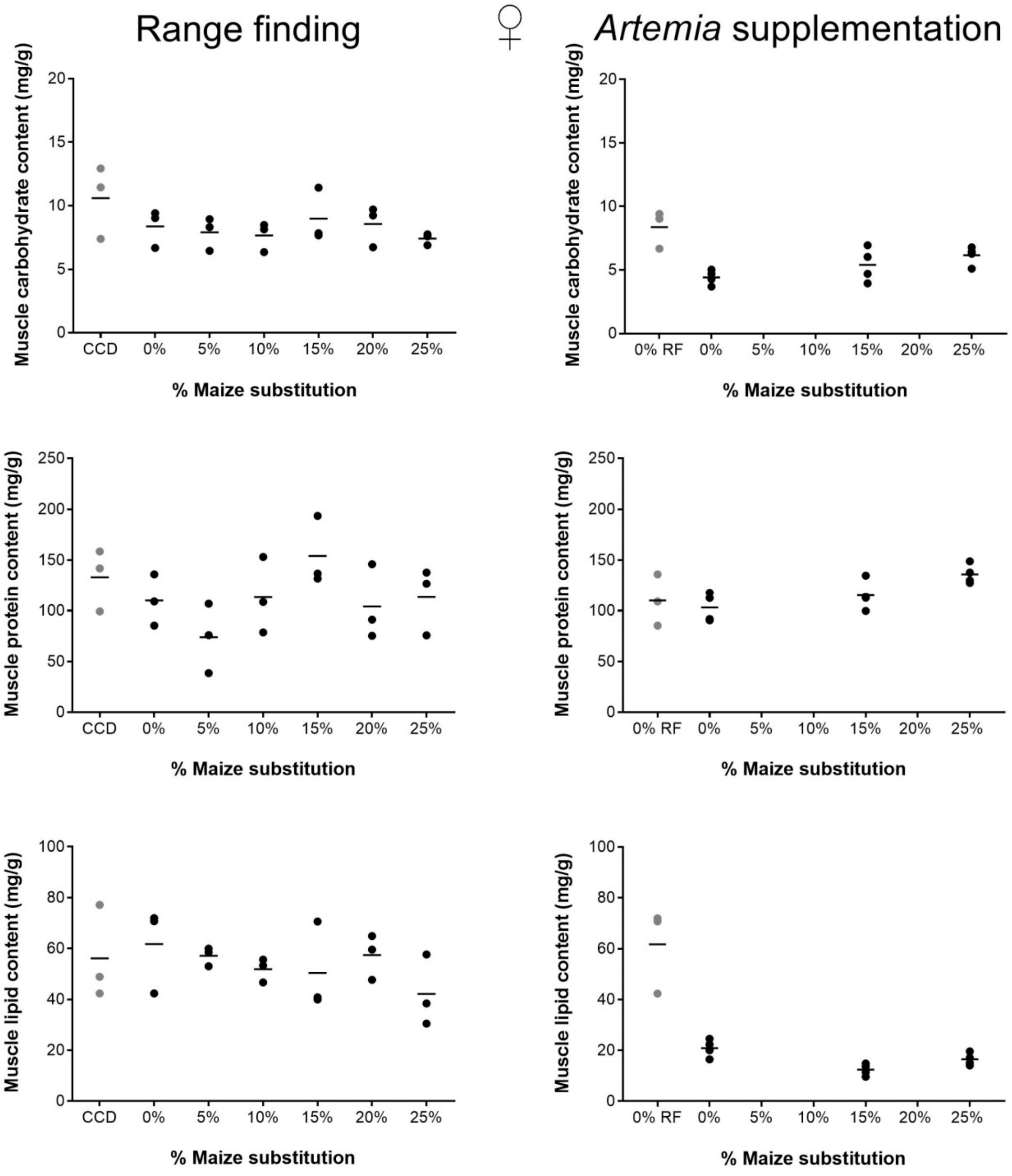

Figure S1.6: Concentrations of energy stores (from top to bottom: carbohydrate, protein and lipid content) in female muscle tissue (Range finding: n = 3; *Artemia* supplementation: n = 4). Increasing the dietary maize substitution rate did not statistically affect energy reserves. Circles represent biological replicates; horizontal lines represent the mean of all replicates; CCD: Commercial control diet; 0% RF: 0% maize substitution values from the range finding feeding trial.

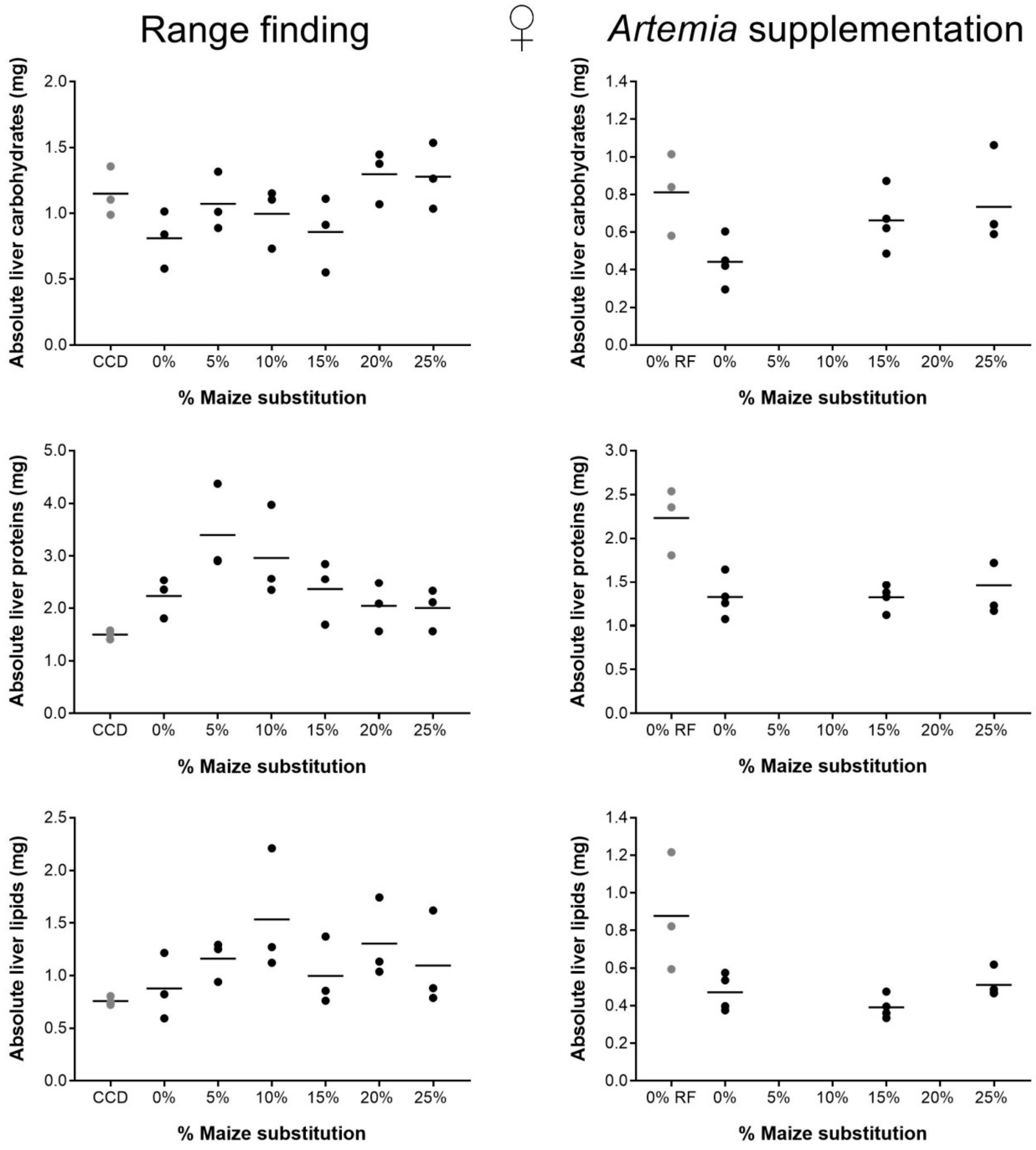

Figure S1.7: Liver carbohydrate, protein and lipid content (from top to bottom) in female liver tissue. Increasing the dietary maize substitution rate did not statistically affect the absolute amount of presented energy reserves (Range finding: n = 3; *Artemia* supplementation: n = 4). Circles represent biological replicates; horizontal lines represent the mean of all replicates; CCD: Commercial control diet; 0% RF: 0% maize substitution values from the range finding feeding trial.

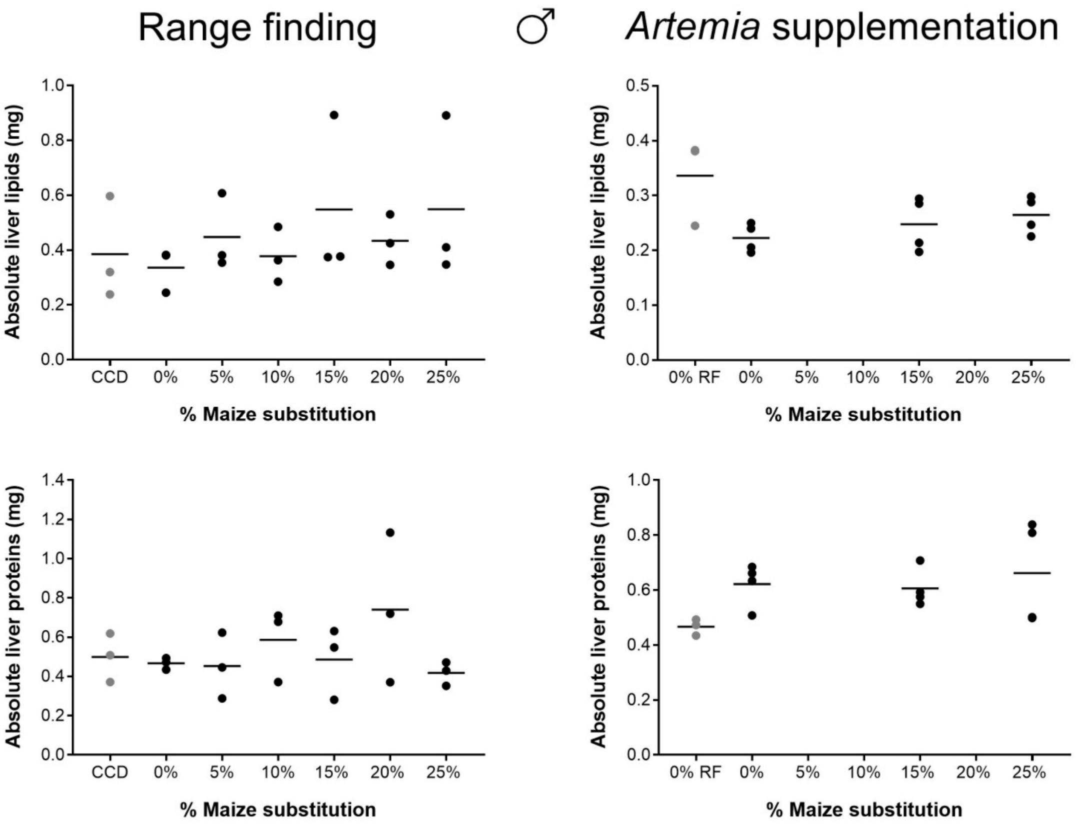

Figure S1.8: Liver lipid and protein content (from top to bottom) in male liver tissue. Increasing the dietary maize substitution rate did not statistically affect the absolute amount of presented energy reserves (Range finding: n = 3; *Artemia* supplementation: n = 4). Circles represent biological replicates; horizontal lines represent the mean of all replicates; CCD: Commercial control diet; 0% RF: 0% maize substitution values from the range finding feeding trial.

S2. RNA purity and integrity and statistical processing steps of raw microarray data

The purity and integrity of the RNA was assessed using a NanoDrop ND-1000 spectrophotometer (Thermo Fisher Scientific, Wilmington, DE) and Fragment Analyzer™ Automated CE System, (Advanced Analytical Technologies, Inc). All samples had minimal OD_{260}/OD_{280} absorption ratios of 1.8, minimal OD_{260}/OD_{230} of 2.0 and minimal RIN (RNA integrity number) of 9.4.

Spots with FG < BG + 2SD (FG: foreground, BG: background, SD: standard deviation of the local background of the entire array) were excluded from the analysis. The normal-exponential convolution model was used to perform background correction and within-array adjustment was done by Loess normalization. Cut offs were set at False Discovery rate (FDR) < 0.05, multiple testing correction using the Benjamini-Hochberg procedure [61] and |log2FC| > 0.585 (log2 fold change, corresponding to a fold induction of at least 1.5 or -1.5).

Table S2.1: Numbers of differentially expressed gene transcripts identified in liver tissue in response to feeding with 25% of maize compared to 0%. Contrasts were calculated both sex dependently and independently.

Contrast	Total No. differential genes p < 0.05	FDR[1]-range	No. true differential genes based on FDR[1]	No. differential genes with FDR[1] < 0.05
Female 25% vs 0% maize	464	0.76-0.9	46	0
Male 25% vs 0% maize	330	0.76-0.99	3	0
Sex independent 25% vs 0% maize	288	0.013-0.07	267	115

[1]FDR: False Discovery rate.

The total number of significantly differentially expressed genes (p < 0.05) was calculated for: female 25% vs 0% maize, male 25% vs 0% maize and 25% vs 0% maize sex independently (Table S2.1). For the sex dependent contrasts, False Discovery rates (FDRs) were high indicating that most of the differential transcripts are considered to be false positives. There were no transcripts with an FDR lower than 0.05, meaning that the identity of the true differentially expressed transcripts could not be determined. The sex independent contrast revealed 115 significantly different transcripts with FDR < 0.05.

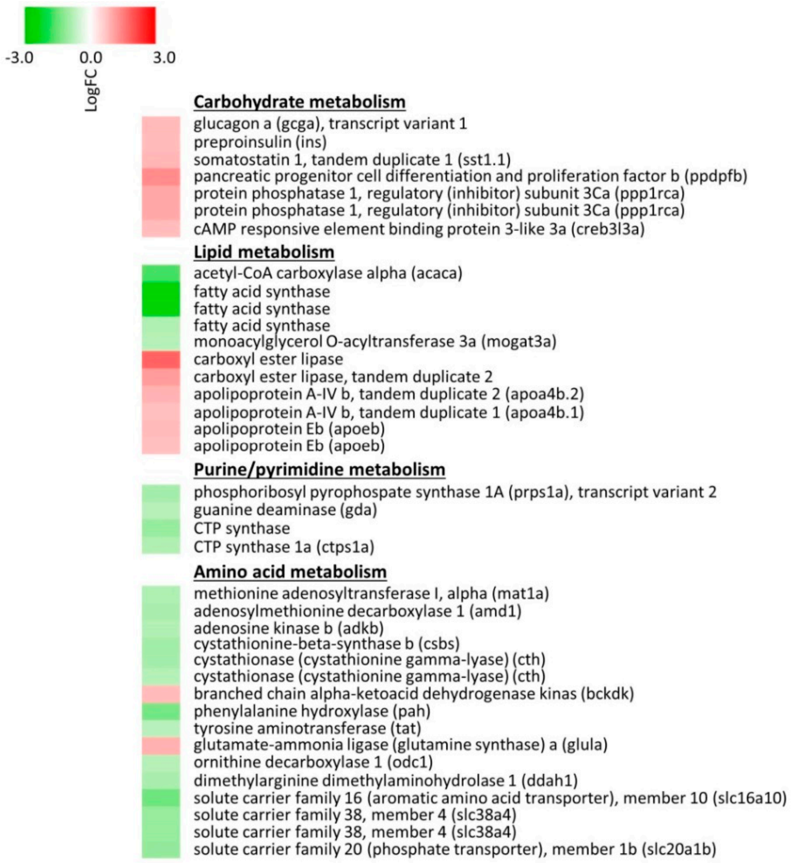

Figure S2.1: Heat map of 31 differentially expressed transcripts depicted in Figure 3,b and c. Green indicates downregulated and red indicates upregulated (False Discovery rate $p < 0.05$).

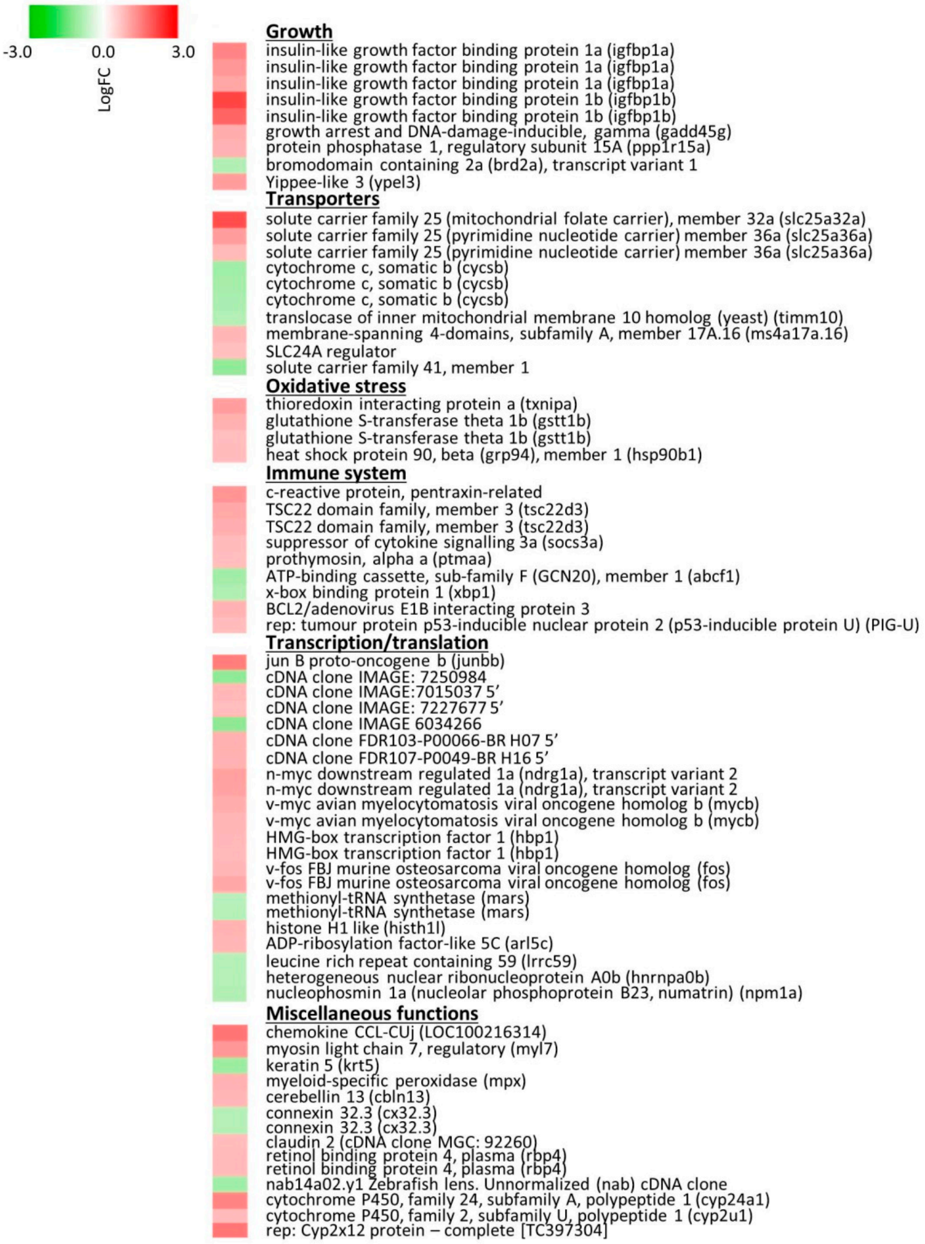

Figure S2.2: Heat map of remaining 55 unique differentially expressed transcripts after feeding with 25% of maize substitution. Transcripts are involved in growth, transport, oxidative stress, immunological, translational/transcriptional processes and miscellaneous functions. Green indicates downregulation and red indicates upregulation relative to 0% maize substitution (False Discovery rate: $p < 0.05$).

S3. Mycotoxin analysis

Twenty-three mycotoxins were simultaneously quantitatively determined using a validated and accredited liquid chromatography coupled to tandem mass spectrometry (LC-MS/MS) method [50]. For aflatoxin B1, stringent maximum levels are set for animal feed (2002/32/EC), while for deoxynivalenol, zearalenone, ochratoxin A, fumonisin B1, fumonisin B2, HT-2 toxin and T-2 toxin non-stringent guidance values for animal feed are described in Recommendation 2006/576/EC and 2013/165/EC. For fumonisin B1 and fumonisin B2, specific guidance values for fish feed are described. However, for the other mycotoxins covered in the Recommendation, no guidance values were set for fish feed. Therefore, the lowest defined guidance value set for animal feed in general was used as a reference.

Table S3.1: Maize cultivation conditions and measured traces of mycotoxins present in the ingredients. The basic mixture comprises all other dry ingredients except for wheat and maize. All values were within the maximum levels and guidance values according to 2002/32/EC, 2006/576/EC and 2013/165/EC.

**List of measured mycotoxins (*legislation*/non-legislation)**		
Aflatoxin B1, **B2, G1 and G2**	*Diacetoxyscirpenol*	Nivalenol
15-acetyldeoxynivalenol	Enniatin B	*Ochratoxin A*
3-acetyldeoxynivalenol	*Fumonisin B1, B2* and B3	Roquefortin C
Alternariol	Fusarenon-X	Sterigmatocystin
Alternariol methylether	*HT2-toxin*	*T2-toxin*
Deoxynivalenol	Neosolaniol	Zearalenon

**Component**		**Mycotoxin**	**Concentration (µg/kg)**	**Guidance value (µg/kg)**
Wheat		Deoxynivalenol	123 ± 35.9	900
		Enniatin B	16 (< cut-off level, 80)	n.a.[1]
		Nivalenol	41 ± 13	n.a.[1]
Basic mixture		Deoxynivalenol	88 ± 26	900
		Enniatin B	28 (< cut-off level, 80)	n.a.[1]
		Fumonisin B1	49 ± 5.8	10 000
		Ochratoxin A	13 ± 1.5	50
Maize	<u>Evaluating maize substitution level:</u> open field, grain maize	Enniatin B	17 (< cut-off level, 80)	n.a.[1]
	<u>Estimating the natural response variation:</u> greenhouse conditions			
	Grain maize MV1	-	-	-
	Grain maize MV2	Enniatin B	21 (< cut-off level, 80)	n.a.[1]
	Silo maize MV3	-	-	-
	Silo maize MV4	-	-	-
	Grain maize MV5	Fumonisin B1	32 ± 3.8	10 000
	Grain maize MV6	-	-	-
	Silo maize MV7	-	-	-
	Silo maize MV8	-	-	-
	Sugar maize MV9	Fumonisin B1	119 ± 14.0	10 000
		Fumonisin B2	65 ± 5.0	10 000
	Energy maize MV10	-	-	-
	<u>Evaluating the use of equivalence testing:</u> greenhouse conditions			
	Experimental GM maize	Fumonisin B1	879 ± 103	10 000
		Fumonisin B2	125 ± 10	10 000
		Fumonisin B3	21 ± 7.6	n.a.[1]
	WT maize	Fumonisin B1	106 ± 12	10 000
	NS maize	Fumonisin B1	535 ± 63	10 000
		Fumonisin B2	54 ± 43	10 000

[1]n.a.: guidance value is not available, no regulation is in place; MV: maize variety; GM: genetically modified; WT: wild type; NS: null segregant.

S4. Supplemental data natural response variation/transgenerational feeding trial

Table S4.1: Summary of endpoint responses for the estimation of the natural response variation (see 4.3.2.).

Endpoints	Sample size (n) per treatment	Mean	SD	Median	Min	Max	Percentile 05	Percentile 95
Relative condition factor W0	60	1.01	0.13	0.99	0.68	1.68	0.83	1.24
Relative condition factor W4	60	1.09	0.14	1.08	0.67	1.55	0.88	1.35
Relative condition factor W8	60	1.14	0.16	1.13	0.59	1.60	0.92	1.43
Relative condition factor W12	60	1.14	0.17	1.12	0.72	2.04	0.90	1.44
Reproduction (96 hpf)								
Fertility (%)	144	87.73	6.98	89.20	74.00	98.80	75.12	98.38
Survival (%)	144	96.88	2.82	97.92	87.50	100.00	88.54	100.00
Hatching (%) – all hatched	144	100.00	-	-	-	-	-	-
Quality of the offspring (96 hpf)								
Swim bladder inflation (%)	144	37.61	11.48	38.30	17.78	68.75	18.12	65.10
Length (mm)	144	4.05	0.17	4.06	2.68	4.45	3.77	4.27
Swimming distance (mm)	144	1370	1148	1066	0	8118	39	3576
Hepatosomatic index %								
Males	30	1.94	0.86	1.81	0.36	7.92	0.90	3.01
Females	30	2.84	0.94	2.82	0.75	7.58	1.35	4.39
Gonadosomatic index %								
Males	3	1.61	0.35	1.52	1.01	2.86	1.11	2.26
Females	3	11.42	2.85	11.22	5.74	21.58	6.66	16.89
Liver energy reserves (mg/g)								
Carbohydrates males	3	44.91	10.83	46.86	22.16	68.66	26.90	66.30
Carbohydrates females	3	38.79	6.21	38.26	22.49	53.77	27.13	53.24
Proteins males	3	46.03	16.09	43.63	23.17	85.80	23.88	78.71
Proteins females	3	63.31	26.11	56.18	36.67	138.00	37.76	137.00
Lipids males	3	81.38	27.38	76.85	27.04	151.90	35.19	141.70
Lipids females	3	112.40	35.46	109.4	46.75	217.6	60.69	172.60
Muscle energy reserves (mg/g)								
Carbohydrates males	3	3.22	0.83	3.18	1.88	5.21	1.98	4.97
Carbohydrates females	3	3.50	0.94	3.53	1.59	5.40	1.62	5.13
Proteins males	3	27.93	3.89	28.54	19.66	34.28	20.68	34.23
Proteins females	3	33.63	6.35	32.61	22.31	50.68	22.36	46.68
Lipids males	3	93.5	25.95	93.55	49.47	141.10	52.03	138.20
Lipids females	3	69.05	27.19	86.81	46.39	140.6	48.32	139.20

Presented values are calculated for the entire dataset of the 10 dietary treatments (3 biological replicates per treatment, 10 male and 10 female fish per replicate), each containing a different non-GM reference maize variety. SD: standard deviation; Min: minimum value; Max: maximum value; W: week; hpf: hours post fertilization.

Table S4.2: The selection of endpoints measured during the transgenerational feeding trial analyzed using traditional statistical testing methods.

**Endpoints**[1]	**Sample size (n) per treatment**	**GM – WT**	**GM – NS**	**NS – WT**	**Ordinary/Mixed one-way ANOVA**	**F test**
Length (F0)	60	0.151	-0.114	0.266	Mixed	$F_{2,6}$=0.73
Length (F1)	60	0.558	0.363	0.195	Mixed	$F_{2,6}$=0.71
Length (F1, 96hpf)	144	0.297	-0.375	**0.675***	Mixed	$F_{2,6}$=3.56
HSI males (F2)	30	-0.482	0.110	-0.591	Mixed	$F_{2,6}$=2.27
Carbohydrates liver males (F2)	3	-1.641	-0.509	-1.131	Ordinary	$F_{2,6}$=3.37
Proteins liver females (F1)	3	**2.047****	0.403	**1.644****	Ordinary	**$F_{2,6}$=22.5****
GSI males (F2)	3	-0.518	-0.255	-0.264	Ordinary	$F_{2,6}$=0.16

[1]All values were standardized after log transformation. The differences of least squares means between the described contrasts are presented. Mixed/ordinary ANOVA was performed and resulting F-values are listed in the final column and shown in bold when they were significant, * $p < 0.05$; ** $p < 0.01$; GM: genetically modified; WT: wild type; NS: null segregant.

S5. Discussion of the equivalence criteria and statistical approach of equivalence testing

The use of equivalence testing requires prior specification of the criteria to be used for demonstrating equivalence. Different strategies for determining equivalence criteria relevant to GM crop safety evaluation have recently been discussed in two key papers: Vahl and Kang [16] and van der Voet et al. [15]. Two main criteria that are discussed in these papers are the scaled average equivalence (SAE, comparing the means of the different diet groups), and the distribution-wise equivalence (DWE, comparing full distributions of the treatments). Further distinction is made among SAE-S(uper), SAE-C(onditional) and SAE-M(arginal) [16]. The SAE-S criterion takes the variation among non-GM reference varieties (i.e., between-reference group variation) into account, while the SAE-C criterion considers both the variation among the non-GM varieties and the variation among replicates (i.e., between-replicates variation). The SAE-M criterion also takes variation among different environmental conditions (or sites) into account. The SAE-M criterion as well as the DWE criteria by Vahl and Kang [16] could not be applied to the data in this work, as they are based on feeding trials executed across different geographic sites.

All equivalence criteria are scaled based on the natural variation among non-GM reference varieties and replicates. In this study, the non-GM reference variation was established in a separate feeding trial (see 4.3.2.). A potential interaction between the respective feeding trials (i.e., the feeding trial for estimating the natural response reference variation and the GM evaluation transgenerational feeding trial) may be taken into account when testing equivalence as an additional source of between-reference group variation. In order to calculate this interaction, a few non-GM maize reference varieties evaluated during the natural response variation feeding trial were also included in the experimental design of the transgenerational GM maize feeding trial. In our analysis, the 'natural response variation x GM evaluation' ('NRV x GM') interaction term between the two feeding trials was included in the SAE criteria, further defined as SAE-Si and SAE-Ci. The DWE criterion equation as developed by van der Voet et al. [15], further defined as DWE_{VDV}, assumes no such interaction term. All 5 criteria that were applied to the transgenerational dataset are outlined in Table S5.1. Conceptually, these criteria express the observed difference in means between the GM maize diet group and the respective control line, either wild type (WT) or null segregant (NS), relative to natural variation in endpoint responses observed among non-GM maize reference varieties and biological replicates of the same treatment.

Table S5.1: Different equivalence criteria applied to the dataset (transgenerational feeding trial).

Criterion	Formula	Description
SAE-S	$\frac{(\mu_T - \mu_C)^2}{\sigma_R^2}$	Only between-reference group variation (σ_R^2) is taken into account.
SAE-C	$\frac{(\mu_T - \mu_C)^2}{\sigma_R^2 + \sigma_E^2}$	Next to between-reference group variation (σ_R^2), the between-replicate variation (σ_E^2) is taken into account as well.
SAE-Si	$\frac{(\mu_T - \mu_C)^2}{\sigma_R^2 + \sigma_{NRV\times GM}^2}$	Similar as SAE-S but with the natural response variation x GM evaluation feeding trials interaction term ($\sigma_{NRV\times GM}^2$) added as additional source of variation to the denominator.
SAE-Ci	$\frac{(\mu_T - \mu_C)^2}{\sigma_R^2 + \sigma_{NRV\times GM}^2 + \sigma_E^2}$	Similar as SAE-C but with the natural response variation x GM evaluation feeding trials interaction term ($\sigma_{NRV\times GM}^2$) added as additional source of variation to the denominator.
DWE_{VDV}	$\frac{(\mu_T - \mu_C)^2 + 2\sigma_{E-GM}^2}{2\sigma_R^2 + 2\sigma_{E-NRV}^2}$	Both between-reference group variation and between-replicate variation are taken into account, where the variation among replicates in the natural response variation feeding trial (σ_{E-NRV}^2) is placed in the denominator, while the variation among replicates in the GM evaluation feeding trial (σ_{E-GM}^2) is placed in the numerator.

µT: mean of the test group, µC: mean of the control group; NRV: natural response variation feeding trial, GM: GM maize evaluation feeding trial; SAE: scaled average equivalence, -S: super/-C: conditional; SAE-Si and SAE-Ci include the interaction term; DWE: distribution-wise equivalence.

Box S5.1: Statistical approach to equivalence testing: algorithm description

Equivalence testing was performed by obtaining the statistical distributions of the equivalence criteria (e.g., van der Voet et al. [15]) and by comparing the equivalence criteria as outlined in Table S5.1 to a critical value in order to accept or reject the null-hypothesis of non-equivalence [16]. To be able to compare the different equivalence criteria that were applied to our dataset (Table S5.1), each of them needed to be transformed to the same scale. Therefore, scaled intervals were obtained for the ratio of means (NS/WT, GM/WT and GM/NS) for all endpoints after log-transformation for all 5 equivalence criteria. Note that as we use log-transformed data, the log of the ratio of the means is equivalent to the differences in the logarithms of the means. For the SAE-S(i) and SAE-C(i), scaling was performed using the standard normal distribution (i.e., dividing the distribution of the equivalence criteria by 1.96) [16]. For DWE_{VDV} [15], the 'equivalence limit scaled difference scale was applied. By doing so, for all equivalence criteria, equivalence is supported if the 95% confidence interval falls within an interval between -1 and 1.

The first step was to set up the statistical models and to obtain parameter estimates and their distributions. Models were specified as outlined in Vahl and Kang [16] for the SAE-S(i) and SAE-C(i) criteria. "Feeding trial" was added as fixed effect for all these models, and the interaction between reference varieties and GM feeding trial was added as random effect for the SAE-Si and SAE-Ci criteria. For DWE_{vdv}, we followed the guidelines for the 'equivalence limit scaled difference scale' as described by van der Voet et al. [15]. For this method, two separate statistical models were set up, one for the natural response variation feeding trial (cf. "historical data") estimating between the variation between reference varieties, and one for the GM evaluation trial (cf. "current data") estimating the differences among WT, NS and GM maize [15].

Next, we obtained the distributions of the different parameters of interest as posterior distributions from Bayesian models with Monte Carlo Markov Chain iterations, using the package MCMCglmm in R (version 3.4.1). For all model parameters, weak prior distributions were assumed and posterior distributions were obtained on the basis of 510000 iterations, after a burn in of 10000 and with a thinning of 50 (a process where only every 50th iteration of the MCMC chain is retained to avoid autocorrelation problems). Thus, all posterior distributions were based on 10000 iterations. The posterior distributions of the SAE equivalence criteria were divided by 1.96 to fit in the limits between -1 and 1 for correspondence with the DWE_{vdv} approach [15].

Figure S5.1 provides an example of one endpoint measured during the transgenerational GM evaluation trial (length, generation F1) to illustrate the different equivalence testing methods based on different criteria calculated.

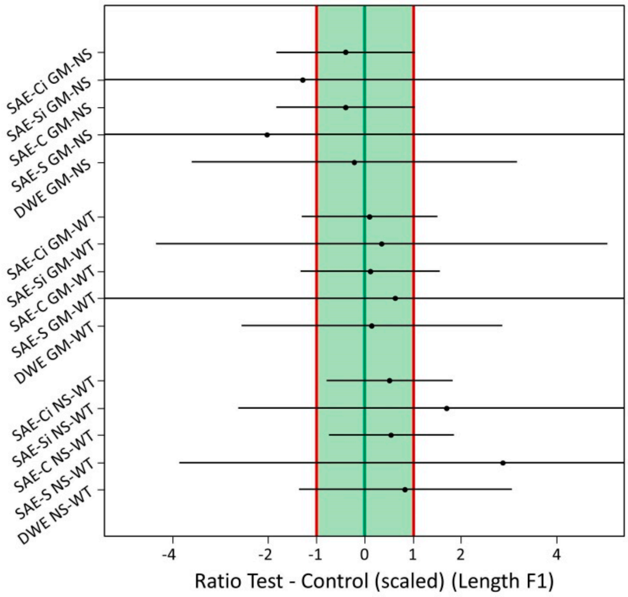

Figure S5.1: The different equivalence testing methods based on different criteria. Different equivalence testing criteria applied to the contrasts GM vs. null segregant (NS), GM vs. wild type (WT) and NS vs. WT for the length of adult fish of generation F1. Mean equivalence limit scaled differences are presented as a black dot, with 95% confidence interval. The vertical red lines represent the equivalence limits (ELs) (-1, +1), calculated based on the non-GM reference variation dataset. SAE: scaled average equivalence, -S: super/-C: conditional; SAE-Si and SAE-Ci include the interaction term; DWE: distribution-wise equivalence.

As the SAE-S criterion only takes the variation among reference groups into account, the use of this criterion is not advised when there is little or no variation observed between the reference varieties [16]. Using the SAE-S criterion, scaled confidence intervals were broad and the central point estimates did not fall within the ELs. This could suggest that there is no 'proof of equivalence' or that equivalence is not 'more likely than not' for the given example (length, generation F1), while the other methods using the SAE-C and DWE criteria suggest that equivalence is 'more likely than not'. Comparison of SAE-S with SAE-C clearly indicates that consideration of the variation among the different replicates is important. When taking replicate variation into account, the scaled confidence limits are narrower and the central point estimates fall within the ELs. When replicate variation is large, as was the case in this example, this is likely to lead to an easier acceptance of equivalence. As

the DWE includes the variation among replicates both in the denominator and in the nominator of the equation, the overestimation of the replicate variation is partially compensated. However, the extent of the compensation will depend on the relative importance of between-replicate variation in the GM evaluation datasets and the natural response variation datasets respectively. In our experiments the importance of the interaction seemed to be limited (Table S5.2). In order to include the interaction in the DWE criterion, a very extensive experimental design would be needed [16].

Table S5.2: Estimated variations for a selection of endpoints measured during the natural response variation and transgenerational feeding trial.

	van der Voet et al., 2017 [15]			Vahl and Kang, 2016 [16]				
	DWE			SAE-S/C		SAE Si/Ci		
**Endpoints**	σ_R^2	$2\sigma_{E-GM}^2$	σ_{E-NRV}^2	σ_R^2	σ_E^2	σ_R^2	$\sigma_{GM\times NRV}^2$	σ_E^2
Length (F0)	0.84	2.11	0.71	0.23	0.15	0.16	0.21	0.11
Length (F1)	0.48	5.66	0.42	0.08	1.98	0.06	0.13	1.92
Length (F1, 96hpf)	0.46	11.1	21.4	0.08	8.04	0.06	0.09	8.08
HSI males (F2)	0.94	2.85	1.44	0.14	0.82	0.13	0.66	0.51
Carbohydrates liver males (F2)	0.69	0.95	0.53	0.48	0.30	0.12	0.49	0.23
Proteins liver females (F1)	0.08	0.24	1.02	0.05	0.43	0.04	**0.06****	0.40
GSI males (F2)	1.36	1.90	0.02	1.50	0.24	0.152	1.50	0.03

σ_R^2 : variation between reference varieties; σ_E^2 : between-replicate variation; σ_{E-GM}^2: between-replicate variation in the GM evaluation feeding trial; σ_{E-NRV}^2: between-replicate variation in the natural response variation feeding trial; $\sigma_{NRV\times GM}^2$: natural response variation x GM evaluation interaction term; SAE: scaled average equivalence, -S: super/-C: conditional; SAE-Si and SAE-Ci include the interaction term; DWE: distribution-wise equivalence; * $p < 0.05$, ** $p < 0.01$.

S6. Feed production protocol

To produce the experimental feed, ingredients were grinded if necessary (Laboratory Mill 3100, Perten) and dry ingredients were mixed together manually; fish oil and Milli-Q water (25 mL per 100 g) were added and the mixture was blended again. The resulting wet dough was subjected to mild extrusion using a pasta appliance to make spaghetti strings of the feed. After air-drying at room temperature for about one week, the strings were grinded to small granular particles (Grindomix GM200, Retsch) and the granules were sieved to obtain the optimal particle size for zebrafish feed: 0.5 mm-1 mm for adult fish and <0.5 mm for growing larvae. All diets were stored in sealed plastic bags at -20°C until use.

S7. Experimental design transgenerational feeding trial and raising the next generation

The transgenerational feeding trial was initiated by feeding a parental generation (F0) with the selected GM/non-GM experimental diets for 12 weeks before producing the first generation (F1). After 6 months of raising, endpoints of F1 were evaluated over 4 weeks after which the second generation (F2) was produced. Again after 6 months of raising, endpoints were evaluated over 4 weeks. A third generation was produced and analyzed until 4 days post fertilization (dpf), but not raised to adulthood. Evaluated endpoints and time points are shown in Figure S7.1.

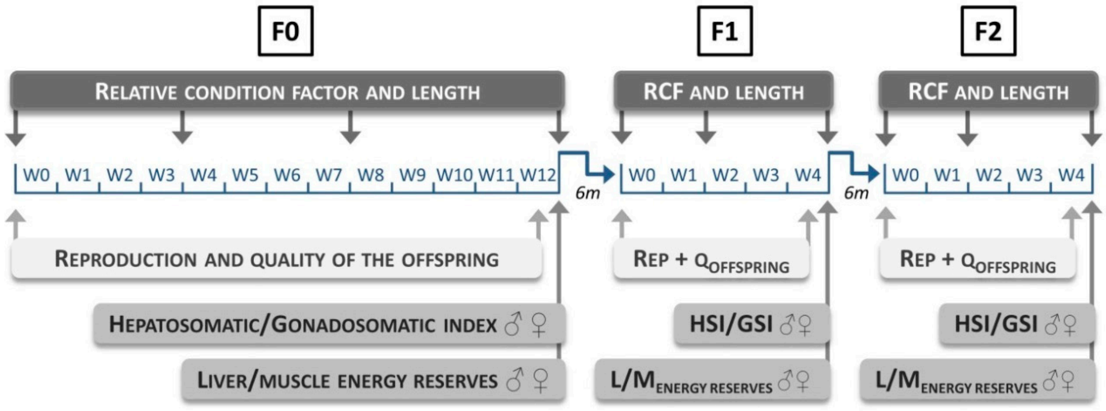

Figure S7.1: Overview of the transgenerational feeding trial. Overview of the selected endpoints, and corresponding time points, measured for the different generations evaluated during the transgenerational feeding trial. RCF: relative condition factor; Rep: reproduction parameters; $Q_{offspring}$: quality of the offspring; HSI: hepatosomatic index; GSI: gonadosomatic index; L/M: liver/muscle tissue; m: months; W: week.

The 3 main diet groups evaluated during the transgenerational feeding trial contained either the GM maize, the WT or the NS maize (15% maize substitution). In addition, 3 non-GM maize variety diet groups were included to calculate the potential interaction between the transgenerational trial and the natural response variation feeding trial (see Supplementary Materials S4). Each diet group consisted of 3 replicates (10 males and 10 females per replicate) and was fed 3 times a day: experimental feed at 9 a.m. and 3 p.m. (2.5% average wet weight per day) and *Artemia* nauplii at 12 a.m. (0.5% average wet weight per day).

Each generation was raised in a semi-static setup, using plastic or glass containers, until stable (no more mortality). The conditions (pH, conductivity, photoperiod, etc.) ideal for adult zebrafish were also applied for breeding larvae (see 4.1). Water volume was increased along with age such that the loading rate decreased with increasing age of the fish. To prevent poor water quality, water was renewed daily. The feeding regime of zebrafish larvae consisted of both dry feed and live feed, and was provided in excess at 4 time points per day, alternating between dry feed and live feed. Two popular live feeds were used: *Paramecia* and *Artemia*. *Paramecia* was used as a live feed for early life-stages (5-15 days post fertilization, dpf) and *Artemia* is used from 10 dpf onwards. Between 5-15 dpf, larvae were fed with a commercial feed (Special Diets Services (SDS)-100 dry food) and with the experimental diets (15% of maize) from 15 dpf onward using a particle size of the feed smaller than 0.5 mm until they reach the age of 3 months.

S8. Energy reserves protocol details

0.2 N perchloric acid (500 μL and 300 μL for liver and muscle respectively) was added to the aliquot (200μL, approximately 8mg tissue) and centrifuged (16 000 g; 2 min; 4°C) to precipitate proteins. The supernatant was aspirated and transferred to a new recipient. Pellets were dissolved in 500 μL 1 N NaOH, incubated for 30 min at 60°C and neutralized with 300 μL of 1.67 M HCl before measuring total protein content. 200 μL of the supernatant was incubated for 30 min at 100°C in 1 mL of Anthrone reagent before measuring the absorption to determine the digestible carbohydrate content [56]. After total lipid extraction [57], 500 μL of 99.99% sulphuric acid was added to 100 μL of the chloroform phase and incubated for 15 min at 200°C.
